# Enhancing Hospital Pharmacy Operations Through Lean and Six Sigma Strategies: A Systematic Review

**DOI:** 10.7759/cureus.57176

**Published:** 2024-03-29

**Authors:** Mohammed Sallam

**Affiliations:** 1 Department of Pharmacy, Mediclinic Parkview Hospital, Mediclinic Middle East, Dubai, ARE; 2 Department of Management, School of ‎Business, ‎International ‎American ‎University, Los ‎Angeles, USA

**Keywords:** dmaic, process control plan, operations management, six sigma, lean leadership‎, satisfaction, service delivery‎, quality metrics, efficiency optimization‎, hospital pharmacy administration‎

## Abstract

Hospital pharmacies are integral to the healthcare system, and evaluating the factors influencing their efficiency and service standards is imperative. This analysis offers global insights to assist in developing strategies for future enhancements. The objective is to identify the optimal Lean Six Sigma methodologies to improve workflow and quality of hospital pharmacy services.

A strategic search, aligned with the Preferred Reporting Items for Systematic Reviews and Meta-Analyses (PRISMA) guidelines, encompassed an extensive range of academic databases, including Scopus, PubMed/Medline, Web of Science, and other sources for relevant studies published from 2009 to 2023. The focus was on management tactics and those examining outcomes, prioritizing publications reflecting pharmacy operations management's state. The quality of the selected articles was assessed, and the results were combined and analyzed.

The search yielded 1,447 studies, of which 73 met the inclusion criteria. The systematic review found a low to moderate overall risk of bias. The number of publications rose during the coronavirus disease (COVID-19) outbreak. Among studies, research output in the United States of America represented 26% of the total. Other countries such as Indonesia, Spain, Canada, China, Saudi Arabia, the United Arab Emirates, and the United Kingdom also made significant contributions. Each country accounted for 12%, 8%, 7%, 5%, 5%, 5%, and 5%, respectively. The pharmacy journals led with 26 publications, and healthcare/medical with 14. The quality category came next with 12 articles, while seven journals represented engineering. Studies used empirical and observational methods, focusing on practice quality enhancement. The process control plan had 26 instances, and the define, measure, analyze, improve, and control (DMAIC) was identified 13 times. The sort, set in order, shine, standardize, and sustain (5S) ranked third, totaling seven occurrences. Failure mode and effects analysis (FMEA) and root cause analysis were moderately utilized, with six and four instances, respectively. Poka-Yoke (mistake-proofing measures) and value stream mapping were each counted three times. Quality improvement and workflow optimization dominated managerial strategies in 22 (30.14%) studies each, followed by technology integration in 15 (20.55%). Cost, patient care, and staffing each featured in three (4.11%) studies, while two (2.74%) focused on inventory management. One (1.37%) study each highlighted continuing education, collaboration, and policy changes.

Analysis of the 73 studies on Lean and Six Sigma in hospital pharmacy operations showed significant impacts, with 26% of studies reporting decreased medication turnaround time, 15% showing process efficiency improvements, and 11% each for enhanced inventory management and bottleneck/failure mode reduction. Additionally, 9% of studies observed decreased medication errors, 8% noted increased satisfaction and cost savings, 6% identified enhancements in clinical activities, 3% improved prescription accuracy, 2% reduced workflow interruptions, and 1% reported increased knowledge. Also, this study has identified key strategies for service delivery improvement and the importance of quality practices and lean leadership.

To the best of the author's knowledge, this research is believed to be the first in-depth analysis of Lean and Six Sigma in the hospital pharmacy domain, spanning 15 years from 2009 to 2023.

## Introduction and background

Introduction

Lean Six Sigma (LSS) initiatives have allowed industrial and service organizations to achieve impressive cost savings, productivity increases, and business sustainability [1]. The ongoing advancements in technology within the healthcare sector have consistently enhanced processes and procedures by applying LSS tools [2,3]. According to Rathi et al. [4], most healthcare LSS studies have focused on management processes, with a noticeable increase in attention from developing nations. Consequently, there has been a shift from focusing solely on preventing and identifying defects and errors to achieving optimization through the digitalization of healthcare and the implementation of "real-time" process control [5]. These advancements can be attained by utilizing the most up-to-date applications of LSS methodologies [6].

Jones et al. [7] conveyed that 70% of any change management initiative might fail without proper measurement. LSS offers consistent measurements by standardizing work and continuously evaluating processes, enabling a structured approach to improvement that prioritizes customer or patient satisfaction in the healthcare domain [8]. These processes consist of recurring, proactive evaluations and enhancements - commonly referred to as the continuous improvement tools of LSS. Also, it prioritizes and captures issues in daily work. LSS tools track progress, sustain initiatives, and prevent fallbacks, and that is why, essentially, LSS drives productive healthcare, business, and patient-centered solutions.

In the global healthcare landscape, hospital pharmacies are critical nodes within the broader medical system, directly impacting patient care and healthcare delivery [9]. The effectiveness of these pharmacies is profoundly influenced by their management strategies, which affect operational efficiency and patient outcomes. As healthcare demands grow and resources become increasingly strained, especially in areas facing economic challenges or healthcare disparities, optimizing pharmacy operations is more critical than ever.

The complexity of managing a hospital pharmacy involves navigating many challenges, from ensuring the safe and timely distribution of medications to effectively managing inventory, staff, and information systems and achieving and maintaining quality accreditations [10,11]. In regions like the Gulf and Middle East, these challenges are compounded by rapid population growth, the prevalence of chronic diseases, and unique cultural and regulatory landscapes. The adoption of innovative and strategic management practices in hospital pharmacies can lead to significant improvements in operational efficiency, which in turn can reduce wait times, lower costs, and ultimately lead to better patient outcomes.

Moreover, with the global rise of international standards and the increasing interconnectivity of healthcare systems, understanding and implementing effective management strategies in hospital pharmacies have implications that exceed regional boundaries.

Background of Lean and Six Sigma methodologies

Lean Methodology

The lean methodology originated at Toyota after World War II and focuses on developing a comprehensive manufacturing system to eliminate waste and promptly meet customer demands [12]. It strives to optimize the flow of value through various processes and emphasizes respecting individuals [13]. Lean defines value as the benefit provided to customers and identifies the main types of waste: excessive work in progress, inefficient processes, and unused employee skills. The successful implementation of lean necessitates active employee engagement and the establishment of a culture of continuous improvement across the entire organization [14]. A lean culture includes ongoing training and development, encouraging employee participation, and maintaining consistent standards [15]. This approach allows adaptability and responsiveness in today's fast-paced environment and provides a straightforward yet efficient problem-solving system [16].

Six Sigma Methodology

Six Sigma is a methodology for improving quality formally, focusing on reducing defects in a process. The term "Six Sigma" indicates that these processes aim to produce a product with no more than 3.4 defects per million cycles, demonstrating high accuracy. Six Sigma employs a structured approach, utilizing data and statistical analysis to evaluate and enhance a company's operational performance. A key component of Six Sigma is its define, measure, analyze, improve, and control (DMAIC) process, an abbreviation for Define-Measure-Analyze-Improve-Control. Initially developed by Motorola in the 1980s, this business strategy applies not only to operational and manufacturing processes but also to transactional and administrative processes. Thus, Six Sigma techniques can improve the quality of business processes, including healthcare operations [17]. The DMAIC process offers a systematic and organized approach to problem-solving, ensuring that issues are addressed and prevented from recurring. It promotes data-driven decision-making and encourages collaboration among team members, ultimately leading to sustainable and practical solutions [12].

Combining Lean and Six Sigma Tools and Strategies

LSS methodologies have been used to reduce or eliminate waste in production processes, clients' waiting time, and product defects. However, little is known about how Lean and Six Sigma can be effectively combined and applied in healthcare management, especially in hospital pharmacy [18]. The combination of Lean and Six Sigma methodologies in healthcare operations, particularly within hospital pharmacies, offers a synergistic approach that optimizes both the efficiency and quality of patient care. The lean methodology focuses on eliminating waste and enhancing workflow processes, while Six Sigma aims to minimize variability and defects through rigorous data analysis. When these methodologies merge, they create a robust framework for achieving operational excellence. This approach ensures that process improvements are directly aligned with the patient's needs, guaranteeing that each step adds value and meets the highest quality standards. By adopting this integrated approach, operations are streamlined, and a culture of continuous improvement is fostered. The goal becomes solving problems as they arise and proactively designing systems that minimize errors. Thus, the combination of Lean and Six Sigma strategies exceeds simply enhancing operations - it strives to transform the culture of hospital pharmacy services, making them as effective, reliable, and patient-centric as possible.

Objective of the Review

This systematic review aimed to thoroughly examine the global practices of Lean and Six Sigma methodologies in hospital pharmacies to identify the most effective strategies in operations management [19]. The essence is categorizing and evaluating efficient methods and instruments for pharmacy operations management incorporating LSS techniques [20-22]. By integrating various approaches, this review aimed to gather a comprehensive source that covers various methodologies and tools used worldwide, focusing on improving pharmacy services to align with complex healthcare processes [23-25].

Considering the healthcare nature of zero tolerance for medication errors, as Khaidir et al. [26] highlighted, the main objective was to provide pharmacies with a definitive guide to improving their operations and enhancing the quality of patient care through the strategic application of LSS techniques, serving as a crucial benchmark and roadmap for optimal operational performance.

## Review

Materials and methods

Research Question

What LSS tools, strategies, and unique operation management approaches were used in hospital pharmacies worldwide to enhance work efficiency and patient service quality? Moreover, what are the main takeaways and the practical consequences of implementing LSS from a managerial perspective?

The review examined the impact of integrating Lean and Six Sigma strategies as operations management tools on hospital pharmacy administration. The question was straightforward, precise, and easily comprehensible.

Search Strategy

The search strategy for this systematic review was carefully developed and executed under the Preferred Reporting Items for Systematic Reviews and Meta-Analyses (PRISMA) guidelines [27]. The research commenced by identifying three primary concepts central to the question: Hospital Pharmacy, Lean Methodologies, and Six Sigma. For Hospital Pharmacy, the search focused on "Hospital Pharmacy*" OR "Pharmacy*" OR "Pharmacy Practice in Hospitals" OR "Hospital-based Pharmacy," aiming to capture the diverse aspects of pharmacy practice within the hospital setting. The second concept, Lean Methodologies, was explored using terms like "Lean principle*" OR "Lean Six Sigma" OR "Lean Process*" OR "Lean think*" OR "Lean Method*" OR "Toyota Lean System*" OR "Lean Practice*" OR “5S Lean” OR "Lean Healthcare" OR "Lean Implementation in Pharmacy", to gather insights into the application of lean principles in improving efficiency and effectiveness in healthcare, specifically in pharmacy operations. Finally, for Six Sigma, the search included "Six Sigma" OR "Six Sigma in Healthcare" OR "DMAIC," focusing on this methodology's specific application in healthcare, particularly in enhancing quality and operational excellence in hospital pharmacies. This comprehensive approach was designed to ensure an extensive understanding of how these methodologies contribute to hospital pharmacy practice. Boolean operators (AND, OR, NOT) combined keywords into a comprehensive search series [28]. Then, they were tailored to the syntax and requirements of multiple databases. The initial search was conducted in one database (Scopus) to assess the relevancy and range of the results, leading to further refinement of the search terms. The process ensured the capture of the most relevant and comprehensive literature available. The search strategy for each database is demonstrated in Table 1.

**Table 1:** Database complete search strategy

Database	Search strategy
Scopus	TITLE-ABS-KEY (Lean AND six AND sigma AND in AND healthcare) AND PUBYEAR > 2009 AND PUBYEAR < 2023
PubMed/MEDLINE	(((Pharmacy[Title/Abstract]) AND (Lean[Title/Abstract])) AND (Six Sigma[Title/Abstract])) OR (Six Sigma[Title/Abstract])
Web of Science (WoS)	((((AB=(Hospital Pharmacy)) OR AB=(Healthcare)) AND AB=(Lean Six Sigma)) OR AB=(DMAIC)) OR AB=(Lean Healthcare‎)

This thorough and systematic approach was created to fully encompass worldwide perspectives on the strategies used in managing the quality of pharmacy operations, which played a significant role in promoting excellence and improving results in hospital pharmacies.

Information Sources

The databases for this systematic review included Scopus, PubMed/MEDLINE, and Web of Science (WoS). To ensure a thorough search, an additional search engine, Google Scholar, was employed.

Inclusion and Exclusion Criteria

For inclusion, the study considered peer-reviewed empirical research articles that provided data on operations management strategies in hospital pharmacies. Distinct from direct clinical treatment results, these studies needed to investigate and provide precise data on management strategies in hospital pharmacy settings, including waste elimination, technological integrations, process and workflow optimizations, staffing models, quality and medication safety improvement initiatives, and policy changes. They were required to report on outcomes related to operational efficiency metrics such as medication error rates, inventory turnover, prescription processing time, medication utilization, and cost reduction. Furthermore, they were required to furnish information about measurable indicators of patient service excellence, including the duration patients waited, the ease of accessing services, surveys assessing patient satisfaction, and the effectiveness of communication between staff and patients. The geographical scope was global with no restrictions, and only studies were published within the last 15 years, from January 2009 to December 2023. Moreover, only English-language articles were considered.

Exclusion criteria included non-empirical studies such as editorials, opinion pieces, technical reports, and commentaries. Studies that did not address hospital pharmacy activities, lean methodologies, or Six Sigma applications in pharmacy healthcare settings were also excluded. Furthermore, non-peer-reviewed sources, preprints, and articles in the non-English language were not considered.

Review, Selection Process, Data Extraction and Synthesis

The selection process for this review followed the structured PRISMA flow diagram, beginning with collecting records from various databases and sources. EndNote 20, a reference management tool, was utilized for the organization and evaluation of titles and abstracts, and it also aided in detecting duplicate entries. Duplicates were first removed, followed by a careful screening by the author for titles to include articles with Pharmacy AND Lean OR Six Sigma. Two independent field expert reviewers evaluated abstracts for the included articles to determine the relevance of each article. Disagreements were resolved through discussion. The potentially relevant articles then underwent a full-text review by the author for final inclusion, with clear documentation provided for any excluded articles.

In parallel, data from the included studies were carefully extracted using an Excel sheet, capturing critical details such as the study design, specific management strategies employed, and outcomes for pharmacy operations and patient care. The data were then narratively synthesized, summarizing the main findings and identifying trends regarding operations management tools and strategies across hospital pharmacies worldwide. This approach ensured a transparent and thorough analysis, offering a detailed understanding of the various management strategies and their implications within the global context of hospital pharmacies.

Quality Assessment

The research articles included in the study were analyzed using the risk of bias in non-randomized studies of interventions (Robins-I) and risk of bias 2 (ROB 2) tools, respectively [29-31]. Cohort and case-control studies were evaluated with the Newcastle-Ottawa Scale, which assesses the selection of study groups, the comparability of the groups, and the ascertainment of the outcome or exposure [32]. These tailored tools and rigorous assessment ensured a systematic and standardized evaluation of the risk of bias and overall quality, which is crucial for accurately interpreting the systematic review results and including reliable and high-quality articles, strengthening research findings.

Data Analysis

The gathered data were compiled in a summary table, and subsequently, manual content analysis was employed for the classification, mapping, and graphics for the findings using Microsoft Excel 2021 [33].

Results

**Figure 1:**
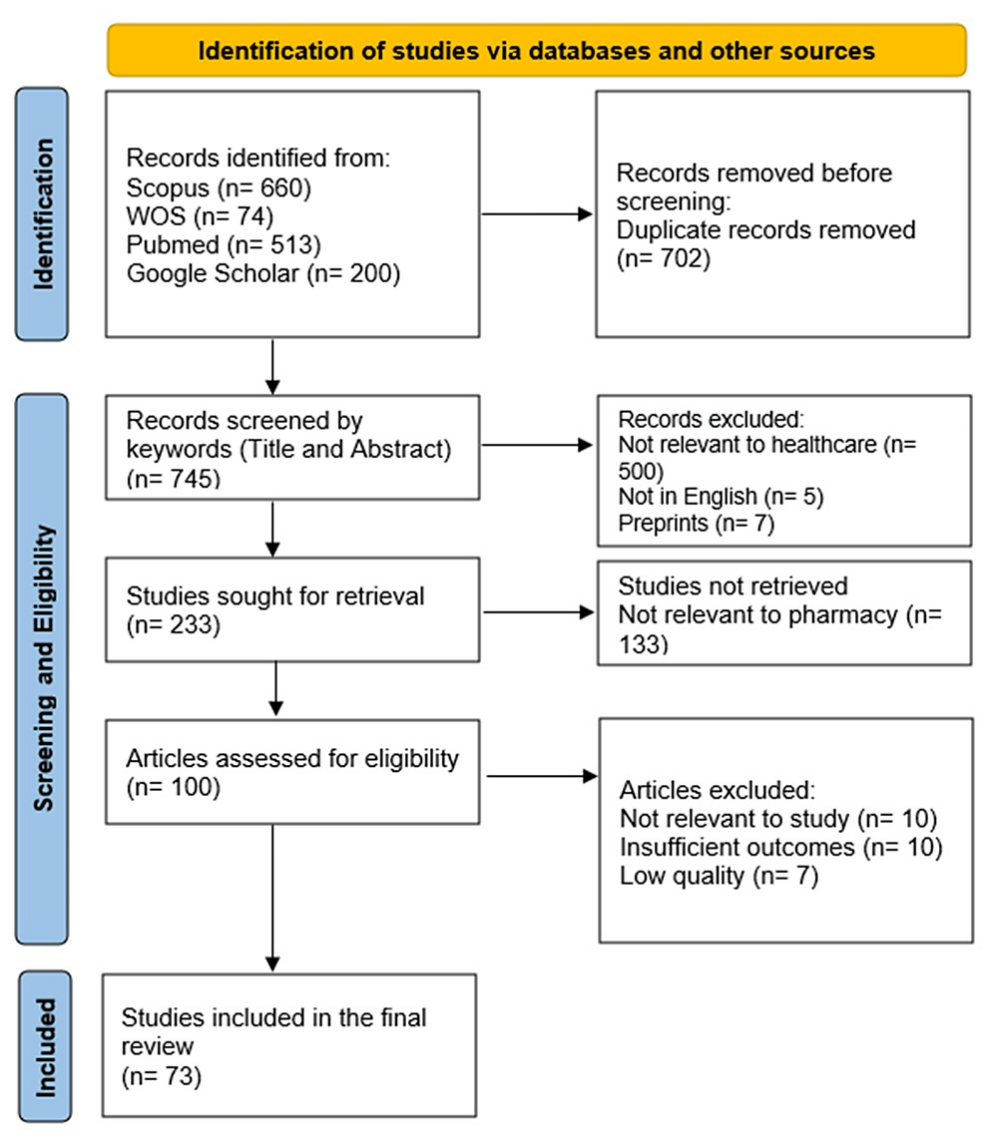
PRISMA 2020 flow diagram for the selection process in the systematic review PRISMA: Preferred Reporting Items for Systematic Reviews and Meta-Analyses

A total of 1,447 files were identified from searching databases, and following the full screening activity, a total of 73 records were eligible to be included in the review. The record selection process is outlined in the PRISMA flowchart provided in Figure 1.

Overview of Included Studies

This summary provides a full description of each study included in the systematic review, detailing important aspects such as the study's reference, publication year, geographical setting, study design, data collection methods, objectives, and hospital size. The comprehensive overview presented the research and management strategies, interventions conducted, metrics used, measured indicators, outcomes evaluated, and results for each study. In addition, the author suggested measures and conclusions to enhance the breadth and depth of the research, establishing a solid comprehension of the context and specific contributions of all the studies included. Table 2 concisely organizes and presents this array of information for quick reference and comparison.

**Table 2:** Characteristics and outcomes of included studies BMC: BioMed Central; FMEA: Failure mode and effects analysis; RPN: Risk priority number; DMAIC: Define-measure-analyze-improve-control

Author	Country	Journal name	Year	Study details	Study objective	Relevant key terms	Management strategy/interventions performed	Outcome measured	Results/findings	Author's recommendations
Kojima et al. [34]	Japan	BMC Health Services Research	2023	Observational study with system analysis conducted at the inpatient medication dispensing unit for oral and topical medicines in Osaka University Hospital, Japan, from February 2017 to July 2020	To identify mechanisms for interruptions in hospital pharmacy work and assess implemented measures for reducing them using a synthetic approach based on resilience engineering and systems thinking	Hospital, Pharmacy, Resilience, Engineering, Performance, Adjustment, Systems, Thinking	Implementation of a medication dispensing tracking system, request-based extra medication delivery, and pass boxes for medicine pickup	Reduction in workflow interruptions (telephone inquiries and counter services) in the hospital pharmacy	Significant reduction in interruptions: from 43 to 18 for telephone calls and from 55 to 15 for counter services, resulting in a 60% reduction in total interruptions	Develop proactive safety management in dynamic work environments such as hospital pharmacies to address systemic problems like interruptions
Abbassi et al. [35]	Tunisia	European Journal of Hospital Pharmacy	2023	Observational prospective analytical study of risk management in the Pharmacy Charles Nicolle Hospital, Tunis, from January 2020 over five months	To improve the medication management process using Failure Mode and Effect Analysis (FMEA) and propose a simplified rating system	Hospital, Pharmacy, FMEA, Risk Management	The application of FMEA is to identify and prioritize vulnerabilities in the medication management process. Proposal of a simplified risk rating system	Identification and reduction of high-priority failure modes in medication management	Twenty-four failure modes were identified, with the highest RPNs being data error in drugs reception (RPN=432), break in the cold chain (RPN=320), and non-optimal pharmaceutical analysis (RPN=280). Good concordance (κ=0.795) between the classic FMEA and the proposed rating system	Implement FMEA for proactive risk assessment and continuous improvement in medication management in hospital pharmacies to enhance patient safety.
Sallam and Snygg [36]	United Arab Emirates (UAE)	Healthcare	2023	Retrospective descriptive study at Mediclinic Welcare Hospital, Dubai, UAE, for 19 consecutive months from January 2021 to July 2022	To assess the impact of the Lean Six Sigma (LSS) methodology on a hospital-wide Antimicrobial Stewardship Program (ASP)	Hospital, Pharmacy, Lean Six Sigma (LSS), DMAIC, CTQ, Stewardship	Implementing Lean Six Sigma methodology and critical-to-quality (CTQ) data collection pre/post interventions, including leadership support, guideline implementation, and audits. ​	Antimicrobial usage, expenditure, compliance, patient outcomes Decrease in hospital’s parenteral antimicrobial expenses, reduction in antimicrobial usage, drop in defined daily dose per 100 bed-days, compliance with surgical prophylaxis bundles, antimicrobial protocols, hand hygiene, and other ASP critical-to-quality metrics​	81.7% decrease in hospital’s parenteral antimicrobial expenses, 54.2% reduction in antimicrobial usage, average defined daily dose per 100 bed-days dropped by 12.5%, improved compliance with surgical prophylaxis bundles	Implement the LSS methodology in healthcare settings to optimize antimicrobial use and enhance patient-centered care, focusing on sustainability through continuous monitoring and ongoing education.
Lourdu et al. [37]	India	Indian Journal of Pharmacy Practice	2023	A prospective observational study conducted in the department of pharmacy at a major trauma care center in Tamil Nadu, India, for Six months from (April 2021 to October 2021)	To improve the pharmacy inventory management system at a major trauma care center using the Six Sigma DMAIC methodology	Hospital, Pharmacy, Six Sigma, DMAIC	Application of the Six Sigma DMAIC (Define-Measure-Analyze-Improve-Control) methodology in the pharmacy inventory management system	Effectiveness of the pharmacy inventory management system, error reduction in different pharmacy facets, and overall process improvement	The study observed significant improvements in inventory management, error reduction across various pharmacy facets, and enhanced operational efficiency.	Implement Six Sigma DMAIC methodology in healthcare pharmacy settings to enhance inventory management efficiency, reduce errors, and improve overall process efficacy in pharmacy services.
Supatmanto et al. [38]	Indonesia	BIO Web of Conferences	2023	A qualitative design includes in-depth interviews, document analysis, and focus group discussions in the Hospital from March 2023 to May 2023	To address inefficiencies in the pharmaceutical supply procurement process, particularly in planning and procurement within the hospital's internal team	Hospital, Pharmacy, Lean Six Sigma (LSS), Procurement Process, ABC, VEN, Analysis	The intervention involves applying Lean Six Sigma up to the "improve" phase, focusing on operational procedure standards, efficiency indicators, and the utilization of ABC/VEN (vital, essential, non-essential) as a tool in the procurement process.	The study measures the effectiveness of proposed changes to the procurement process, emphasizing the reduction of waste and overproduction in pharmaceutical supply planning.	Findings highlighted the lack of established procedures and efficiency benchmarks, leading to prolonged procedures and low internal team communication.	Utilize ABC VEN and efficiency indicators to tackle waste in the supply planning process and establish new standard operating procedures to enhance efficiency and communication.
Almalki et al. [39]	Saudi Arabia	Global Journal on Quality and Safety in Healthcare	2023	A quality improvement project will be conducted using the Six Sigma approach, FMEA, and King Fahad Armed Forces Hospital staff surveys. Baseline assessment started in December 2019, interventions began in January 2020, and primary aims were set for the end of 2021	To improve medication inventory management and error prevention by implementing an automated system to reduce dispensing errors and improve efficiency	Hospital, Pharmacy, Six Sigma (FMEA), Automation	Implementation of automated dispensing cabinets and reengineering workflows, using Six Sigma and Failure Mode Effect Analysis (FMEA)	Reduction in medication turnaround time, decreased stock items, reduced medication consumption	83% reduction in turnaround time, 24% reduction in monthly medication consumption, 72% reduction in returned items, USD 4,100,000 annual savings from expired medications	Implement automated dispensing systems in hospitals to significantly enhance efficiency, reduce errors, and achieve substantial cost savings in medication management.
Wang et al. [40]	China	BMC Health Services Research	2022	Quality improvement study applying information-intelligence technologies in Pharmacy Intravenous Admixture Services (PIVAS), data collected over a period from July to September 2019 and 2020	To improve efficiency and reduce error risks in Pharmacy intravenous admixture service (PIVAS) through the application of information-intelligence technologies	Hospital, Pharmacy, Information-Intelligence Technologies, Prescription Review, Increase Efficiency, Reduce Errors, Robots	Establishment of an intelligent prescription-checking system application of intelligent equipment in PIVAS (automatic labeling machine, intravenous compounding robots, intelligent infusion bag sorting system, intelligent logistics robots)	Work efficiency and error risk in intravenous admixture service	Marked improvement in prescription checking and intervention success rates; reduced error risk with intelligent devices. Some devices need enhancements	Emphasize the continual improvement of information-intelligence technologies in PIVAS for better efficiency and safety
Patel and Quinn [41]	United Kingdom (UK)	BMJ Open Quality	2022	A service quality improvement project using Plan-Do-Study-Act cycles; data collection from the electronic prescribing system; Sheffield Teaching Hospitals NHS Foundation Trust; Duration not specified.	To speed up medication delivery after placing a request on the e-ordering system to reduce delays in administration	Hospital, Pharmacy, Plan, Do, Study, Act (PDSA)	Implementation of plan-do-study-act (PDSA) cycles to improve critical medication supply. Implementation of a critical medication checklist, optimization of the e-ordering system	Availability of critical medications on the ward within 30 minutes of the next scheduled administration	Improvement from 85% to 93% in the availability of critical medications on the ward within 30 minutes	Continue to refine and optimize the e-ordering system and critical medication supply processes for better efficiency.
Huang et al. [42]	China	European Journal of Hospital Pharmacy	2022	5S Quality improvement project in the outpatient-emergency pharmacy at a local hospital	To assess the effects of 5S management on pharmacy quality and staff capacity in outpatient emergency services	Hospital, Pharmacy, 5S, Lean, Management, Waiting, Time	Implementation of 5S management in outpatient-emergency pharmacy	Service quality and staff capacity	The working area increased from 45.16 m^2^ to 51.64 m^2^, resulting in a 16.8% elevation of space utilization rate. Improved dispensing accuracy (The drug dispensing error rates were 45.6% lower), (36.9% and 37.9% lower for the waiting time of patient and drug dispensing time, respectively) increased satisfaction and enhanced pharmacist performance (The satisfaction rates of patients and pharmacists were increased by 41.8% and 80.4%, respectively)	Implement 5S management to improve quality and efficiency in hospital pharmacies effectively.
Kameo et al. [43]	Brazil	Journal of Contemporary Nursing	2022	Experience report on the implementation of the 5S Program in a hospital pharmacy service, conducted from January 20 to May 31, 2021, at a women's healthcare hospital in Campinas, São Paulo, Brazil	To describe the implementation of the 5S Program in a hospital pharmacy service	Hospital, Pharmacy, 5S, Lean, Methodology	5S Program in hospital pharmacy	Cleaner and more organized workspaces, reduced waste, improved inventory control, improved employee motivation and collaboration, and enhanced patient safety in medication handling and dispensing.	Qualitative improvements are described and visually displayed.	Implement 5S program in healthcare pharmacy settings to improve management efficiency, workspace organization, and quality of healthcare services.
Alipour et al. [44]	Iran	Journal of Health Organization and Management	2022	Experimental study conducted from 2015 to 2018 in a 300-bed hospital and regional healthcare centers affiliated with the Petroleum Industry Health Organization	To provide a model for strategic management of pharmacy departments using the Balanced Scorecard (BSC) framework	Hospital, Pharmacy, BSC, Framework	BSC framework focuses on finance, patient satisfaction, internal processes, and learning/growth. Implementation of various measures such as protocols for expensive medications, staff training, and coding medical equipment	Financial performance, patient satisfaction, operational efficiency, resource utilization	Significant increases in patient satisfaction and gross profit; reduced costs in various departments; saved USD 539,137 and USD 442,899 through specific interventions	Implement the BSC framework in healthcare settings to improve financial performance, patient satisfaction, and operational efficiency.
Chen et al. [45]	Taiwan	Socio-Economic Planning Sciences	2022	Dynamic network data envelopment analysis applied to hospitals and other pharmacies. Data from 2014–2019 from Taiwan's cities, the Ministry of Health and Welfare, and the Bureau of National Health Insurance	To assess the divisional efficiency of hospital and pharmacy utilization and investigate dynamic changes and spatial differences for more productive utilization of healthcare services	Hospital, Pharmacy, Efficiency, Analysis	Assessing hospital pharmacy via Dynamic Network Data Envelopment Analysis	Efficiency of hospitals and pharmacies	The mean efficiency scores for overall healthcare, hospital, and pharmacy among cities were 81.9%, 89.2%, and 76.7% respectively	Focus on addressing inefficiencies in hospital and pharmacy divisions and track productivity changes over time.
Aan Adriansyah et al. [46]	Indonesia	To Maega: Journal of Community Service	2022	An empirical study involving Lean management training with a Duration of one day (20 June 2022)	To provide Lean Management training in the pharmacy unit of the hospital to enhance effective and efficient governance, thereby improving service quality and profitability	Pharmacy, Lean, Management, Efficiency	Implementing Lean management training involving 18 workers, focusing on enhancing service efficiency. Training included lectures, discussions, and simulations.	Enhancement in knowledge and understanding of Lean Management principles among participants	Increase in participants' knowledge by 18%	Conduct Lean Management Training to make services more effective and efficient, thus improving service quality and increasing profitability.
Imran et al. [47]	Indonesia	Journal of Industrial Engineering and Halal Industries	2022	A case study using action research methods with qualitative and quantitative approaches, conducted from February to April 2019 at Hospital	To increase efficiency in prescribing compounded medicines and reduce waiting times for patients at Hospital ABC Purbalingga using a Lean approach	Pharmacy, Lean, Management, Waiting, Time	Implementation of Lean management tools such as value stream mapping, 5-why’s analysis, and identification of value-added and non-value-added activities	Efficiency of pharmaceutical services, reduction in waiting time for patients, optimization of process flow	Reduction of non-value-added activities by 26.14%, optimization of process flow with 49.64% value-added activities post-implementation	Implement Lean management practices to enhance pharmacy service efficiency and reduce patient waiting times.
Al Nemari and Waterson [48]	Saudi Arabia	JMIR Human Factors	2021	A retrospective review of a Six Sigma performance improvement project with the introduction of automation in an outpatient pharmacy department was conducted at King Fahad Medical City. The study lasted 20 months (April 2019-December 2020), with a go-live for the automated pharmacy after three months (July 2019)	To measure the impact of automation integration on service safety and efficiency, staff reallocation and reorientation, and workflow in the outpatient pharmacy department	Hospital, Pharmacy, Six Sigma, SS, Automation	Introduction of robotics and automation, Six Sigma methodology, process mapping, turnaround times, discrete event selection for improvement	Patient time in the department, dispensing error rate, completeness of prescriptions, wastage cost	- Wrong patient wrong medication errors reduced by 5.25:1 - Inadequate counseling errors reduced by 2.5:1 - Incomplete prescriptions decreased from 3.0% to 1.83% - Dispensing error rate dropped from 1.00% to 0.24% - Medication turnaround time reduced by 83% - Monthly medication consumption decreased by 24% - Ward stock reduced by 81% - Returned items decreased by 72% - Telephone calls processed improved by 160%	Implement automation and Six Sigma for improvement in hospital pharmacy.
Trakulsunti et al. [49]	Thailand	International Journal of Quality and Reliability Management	2021	Action research study implementing Lean Six Sigma at an inpatient pharmacy in a Thai teaching hospital. Duration: April 2018 - August 2019	To illustrate the use of Lean Six Sigma (LSS) and its associated tools to reduce dispensing errors in an inpatient pharmacy of a teaching hospital	Hospital, Pharmacy, Lean Six Sigma (LSS), (DMAIC), Process, Improvement	Lean Six Sigma DMAIC methodology; focus on process improvement and error reduction	Reduction in Dispensing errors, Process performance, Staff and patient safety	Reduction in dispensing errors from 6 to 2 incidents per 20000 inpatient days per month (66.66% reduction). Improved dispensing process, patient safety, and communication between pharmacy and technicians	Apply Lean Six Sigma and continuous improvement methodologies for enhancing pharmacy services and patient safety
Chen et al. [50]	China	Journal of Evaluation in Clinical Practice	2021	A prospective exploratory study utilizing Lean Six Sigma and Failure Model and Effect Analysis (FMEA) in a hospital pharmacy department. Analysis was done on dispensing errors between August 2012 and July 2018. Measures improved and impact assessed a year later (Sep 2018 to Aug 2019)	To utilize Lean Six Sigma (LSS) and Failure Model and Effect Analysis (FMEA) to reduce and prevent dispensing errors in the hospital pharmacy	Hospital, Pharmacy, Lean Six Sigma, (LSS), Failure mode and effects analysis (FMEA)	Application of LSS and FMEA methodologies to identify, address, and reduce dispensing errors in pharmacy operations.	Reduction in dispensing errors, identification of high-risk medications, improvement in pharmacy dispensing processes	There was a significant decrease in dispensing error incidence rate from 0.33 cases per 10,000 medication orders to 0.19 cases after the intervention.	Apply LSS and FMEA techniques in healthcare settings to systematically reduce dispensing errors and enhance medication safety.
Hammoudeh et al. [51]	Jordan	Hospital Pharmacy	2021	Lean management implementation study; outpatient pharmacy of a comprehensive cancer center; data collection from pharmacy electronic system and employee satisfaction surveys; conducted from June 2015 to December 2016	To reduce patient waiting time and improve the satisfaction of patients and staff in the outpatient pharmacy	Hospital, Pharmacy, Lean, Management, Process, Optimization, Training, Waiting, Time	Lean management tools (A3 problem-solving report, value stream mapping), staff training, process optimization	Patient waiting time, patient and employee satisfaction	Significant reduction in patient waiting time for prescriptions (22.3 minutes to 8.1 minutes for 1-2 medications, 31.8 minutes to 16.1 minutes for 3+ medications) and improvement in patient satisfaction (62% to 69%)	Implement lean management in healthcare settings to improve operational efficiency and satisfaction among patients and staff
Iswanto [52]	Indonesia	International Journal of Lean Six Sigma	2021	Case study; pharmacy unit in a hospital; data analysis including inventory purchases, hospital deficit, patient length of stay (LOS); periods before and during the COVID-19 pandemic	To assess the impact of Lean Six Sigma (LSS) implementation on hospital profitability and pharmacy unit operations before and during the COVID-19 pandemic	Hospital, Pharmacy, Lean Six Sigma (LSS), LOS	Implementation of Lean Six Sigma methodologies in the pharmacy unit; evaluation of hospital profitability, inventory management, and patient LOS	Reduction in inventory purchases, hospital deficit changes, patient LOS, and hospital performance	Post-LSS implementation showed a decrease in inventory purchases (27% pre-pandemic, 29% during the pandemic, 37% new normal) and a reduction in hospital deficit (26% pre-pandemic, 10% during the pandemic)	Explore Lean Six Sigma in various hospital units to improve efficiency and adapt to pandemic challenges; assess the long-term sustainability of LSS in changing healthcare environments.
Martín-Conde et al. [53]	Spain	Farmacia Hospitalaria	2021	The prospective observational single-center study, implementing Lean methodology through patient participation. Conducted in the outpatient pharmacy at a hospital in Barcelona. Actions were taken in late 2019-mid 2020. Waiting time was tracked monthly, and satisfaction was measured with questionnaires in Dec 2019 and Dec 2020	To improve the dispensing process and pharmaceutical care quality in the outpatient pharmacy through patient participation and Lean methodology, analyzing efficiency and patient satisfaction.	Hospital, Pharmacy, Lean, Methodology, Satisfaction, Waiting, Time	Implementation of Lean tools and methods, patient focus groups, patient satisfaction surveys	Patient satisfaction, efficiency of pharmacy service	Significant improvements in waiting times (from 35% to 4.5% of patients waiting over 30 minutes) and overall patient satisfaction	Utilize patient participation and Lean methodology in outpatient pharmacy services to enhance efficiency and patient satisfaction
Yuliati and Andriani [54]	Indonesia	Open Access Macedonian Journal of Medical Sciences	2021	Operational research with qualitative and quantitative approaches, including direct observation and in-depth interviews, conducted at Grha Permata Ibu Hospital from October to December 2020	To reduce waiting time for prescription services using Lean Kaizen through the Plan-Do-Check-Act (PDCA) approach	Hospital, Pharmacy, Lean, Kaizen, Plan, Do, Check, Act (PDCA), Waiting, Time	The Lean Kaizen method uses the PDCA cycle, focusing on waste elimination in the prescription service process.	Reduction in lead time (LT) for prescription services	Significant reduction in lead time: from 135.31 minutes to 9.11/7.49 minutes in non-concoction prescriptions and from 185.17 minutes to 31.09/29.15 minutes in concoction prescriptions	Explore and implement Lean Kaizen with the PDCA approach in healthcare services to enhance efficiency and reduce patient waiting times
Sadi et al. [55]	United Arab Emirates (UAE)	International Journal of Healthcare Management	2021	Quality improvement project in polyclinic pharmacy at Tawam Public Hospital in the United Arab Emirates to improve patient experience and satisfaction with pharmacy services	To improve waiting time in polyclinic pharmacy	Hospital Pharmacy, HIS, Quality, Improvement, Waiting, Time	Workflow change and optimal utilization of the hospital information system (HIS)	The difference in prescription processing time between pre- and post-workflow process changes for both advanced and routine prescriptions	Reduction of waiting time from 21.5 to 4 min as per Qlik View Customer Satisfaction tool. A patient satisfaction survey suggested that 82% of patients were either extremely satisfied or satisfied with the reduced waiting time	Improve workflow and optimize HIS to achieve a reduction in total patient waiting time, improve patient satisfaction and overall experience
Owusu-Guha et al. [56]	United Staes of America (USA)	INNOVATIONS in pharmacy	2021	Implementing Lean methodology in clinical pharmacy services, including using Key Performance Indicators (KPIs) and Gemba walks. Conducted at OhioHealth Riverside Methodist Hospital, a community teaching hospital	To improve critical care clinical pharmacist productivity by developing and implementing clinical metrics using Lean methodology	Hospital, Pharmacy, Lean, Methodology, Key, Performance, Indicators (KPIs), Improvement, Gemba, Walks, Improvement	Application of Lean principles, development of clinical KPIs, Gemba walks for process observation and improvement.	Effectiveness of KPIs in improving critical care clinical pharmacist productivity	Critical Care Pharmacy KPI Metrics were created to cover service delivery, safety, quality, productivity, and cost management. In 90 days, ICU medication reconciliation improved with increases in all units, including a 50% rise in the medical ICU and neurological critical care unit. Surgical ICU doubled. 89 hours of pharmacist time were dedicated to reconciliation, taking an average of 11.13 minutes per event	Utilize Lean principles and methodologies to enhance clinical pharmacy services and improve pharmacist productivity.
Kallal et al. [57]	United States of America (USA)	BMJ Open Quality	2020	Quality improvement process using Lean Six Sigma (LSS)	To avoid the unintended omission of warfarin, an anticoagulant is used to prevent and treat thromboembolic events, which can lead to serious medical complications. These complications include increased medical costs, hospitalizations, and significant patient harm, including increased risk of thrombosis and mortality	Hospital, Pharmacy, Lean Six Sigma (LSS), EHR	A system alert was implemented in the electronic health record to alert providers of patients who received warfarin during admission.	Reduction in patients discharged without intended warfarin prescription	Reduction from 10.5% (4/38) to 0% (0/40) in omitted warfarin prescriptions post-intervention. Alert tracking enhanced the ability to identify patients at risk for warfarin omissions. Process sustainability has been achieved by embedding system alerts in the electronic health record to trigger process steps	Implement system alerts in electronic health records to reduce medication omissions; further research may explore similar strategies for other critical medications
Iswanto and Rosady [58]	Indonesia	Systematic Reviews in Pharmacy	2020	Empirical Study Lean implementation assessment in pediatric pharmacy at Mother and Child Hospital, 20 outpatient policlinics, 63 beds from May to July 2019	To assess the successful implementation of Lean in the children’s outpatient pharmacy	Hospital, Pharmacy, Lean, Methodology, Cost, Reduction, Efficiency, Improvement	Inventory cost analysis, Cost Reduction, Efficiency Improvement	Inventory cost reduction	Inventory cost reduced from US$ 22,494 to US$ 15,128 monthly; total savings of US$ 22,097 in three months; benefit to cost ratio of 592%	Implement Lean in various hospital pharmacy units, considering medicine availability for unexpected cases.
Bashir et al. [59]	United Arab Emirates (UAE)	International Journal of Industrial and Systems Engineering	2020	Quality improvement project. Application of group technology to drug shelving in public hospital outpatient pharmacy	To improve workflows, reduce the total distances traveled by pharmacy drug pickers, and reduce patient waiting time.	Hospital, Pharmacy, Lean, Drug, Shelving, Technology	Group technology for drug shelving in outpatient pharmacy	Workflow efficiency, distance traveled by drug pickers, patient waiting times	Applied to a public hospital's outpatient pharmacy; 114 drugs grouped into eight cells, optimizing shelf arrangement	Implement practical, simple methods for improving pharmacy efficiency by minimizing drug picker travel distance, retaining group arrangement of drugs
Nina and Hakim [60]	Indonesia	IOP Conference Series: Materials Science and Engineering	2020	Observational study with interviews, application of Lean Hospital methodology, value stream mapping (May and June 2020)	To improve the process of drug service delivery in outpatient pharmacy using Lean principles	Hospital Pharmacy, Lean, FMEA, Waiting, Time	Application of Lean principles, Fishbone analysis, FMEA (Failure Modes and Effects Analysis). Time measurement of drug delivery process, Process cycle efficiency. Reduction in waiting time, Improvement in service delivery	Service time, Process cycle efficiency for concoctions and non-concoctions	Service time of 61 minutes for concoctions and 32 minutes for non-concoctions; Process cycle efficiency of 74% for concoctions and 61% for non-concoctions	Increase human resources in the dispensing section, categorize drug shelves, apply 5S culture, use simulation software
Gao et al. [61]	China	Leadership in Health Services	2020	Action research study. Nine-month action research (September 2016 - May 2017) project observing lean implementation in a pharmacy intravenous admixture service. Jinan Central Hospital is affiliated with Shandong University, Jinan.	To improve operational efficiency in pharmacy intravenous admixture services using lean management principles	Hospital Pharmacy, Lean Management, Operational Efficiency, Admixture	Implementing Lean management principles to optimize Pharmacy Intravenous Admixture Services (PIVAS) center operations.	Efficiency of operations, quality of work, employee satisfaction, and clinical satisfaction rates	The staff in the A shift was reduced from 5 to 4, work start times for A and B shifts were postponed, and work efficiency improved. The number of unqualified dispensing drugs decreased by 76.2%, and clinical satisfaction rates increased from 90% to 95%	Implement Lean management in healthcare settings to enhance operational efficiency, improve employee and clinical satisfaction, and ensure high-quality pharmaceutical services.
Vashishtha [62]	United States of America (USA)	International Journal of Research in Medical and Basic Sciences	2020	Quality improvement using Lean Six Sigma DMAIC Approach at Center for Geriatrics-UNT Health from February 2018 - July 2018	The Lean Six Sigma Approach is used to decrease patient wait time in a geriatric outpatient setting.	Hospital, Lean Six Sigma, LSS, DMAIC, Waiting, Time	Implementation of Lean Six Sigma principles. Reduction in average wait time and improvement in patient flow. Enhanced operational efficiency, improved patient and employee satisfaction	Patient wait time	Average wait time improvement by 10 minutes	Apply Lean Six Sigma for Process Improvement
Sunarko and Koeswo [63]	Indonesia	Indian Journal of Forensic Medicine & Toxicology	2020	Observational empirical study using root cause analysis to identify causes of prolonged waiting times at Private Hospital in Indonesia	To identify and analyze the root causes of prolonged waiting times for compounded prescriptions in the hospital pharmacy	Hospital Pharmacy, Root Cause Analysis (RCA), Waiting, Time	Root cause analysis is a strategic approach that involves hospital directors, pharmacy department heads, and staff. Analysis of waiting times, pharmacy service efficiency	Identification of causes for prolonged waiting times and recommendations for improvement	Identification of critical factors such as drug and human resources shortage, waiting times for drug preparations, and health insurance coverage confirmation duration	Optimize drug management, improve coordination in drug distribution, and redesign the waiting room in the hospital to improve patient satisfaction and efficiency
Caro Teller et al. [64]	Spain	Journal of Healthcare Quality Research	2020	Observational empirical study using Lean Six Sigma DMAIC	To evaluate efficiency improvements in the medication dispensing circuit after the application of the LSS methodology	Lean Six Sigma, LSS, DMAIC, SIPOC, VSM	LSS methodology includes the DMAIC cycle, SIPOC diagram, and Value Stream Mapping (VSM). Metrics involved urgent medication orders per day and the percentage of online orders	Performance improvement in medication dispensing circuit measured in sigma levels and staff time cost savings	Performance increased from 60% to 94% in Thoracic Surgery and 71% to 93% in Cardiology. Cost savings of 798.2 € initially, increasing to 2,228.5 € after 6 months	Implement LSS methodology in healthcare settings to enhance efficiency and reduce costs in medication dispensing processes, focusing on continuous improvement.
Hohmeier et al. [65]	United States of America (USA)	Research in Social and Administrative Pharmacy	2020	Review and Case Study of theories and science of prioritization in healthcare, and a case study involving the development and use of a Medication-Related Problems (MRP) prioritization matrix	To highlight the importance of prioritization in clinical pharmacy practice and present a case for the application of prioritization matrices	Healthcare, Lean Six Sigma, LSS	Utilization of Multicriteria Decision Analysis and Lean Six Sigma to develop a prioritization matrix for MRPs	Improvement in prioritization skills among pharmacists, as evidenced by self-efficacy in assigning priority ratings to MRPs	Significant improvement in pharmacists' self-efficacy post-intervention with the use of the prioritization matrix	Investigate, develop, and provide resources and training to improve pharmacist skills in managing new clinical workloads and incorporate prioritization tools into practice to improve the efficiency and effectiveness of patient care services.
Antony, et al. [66]	Norway	Leadership in Health Services	2019	Exploratory Study, Mixed method approach with surveys and interviews	To explore the utilization of Lean Six Sigma in reducing medication errors in Norwegian public healthcare	Healthcare, Lean Six Sigma (LSS)	Application of Lean Six Sigma methodologies. Analysis of medication error reduction	Effectiveness in reducing medication errors, especially administration errors	Implementation of Lean Six Sigma in its infancy with challenges such as lack of training and awareness	Encourage further research into implementing Lean Six Sigma in healthcare to reduce medication errors.
Creed et al. [67]	Ireland	Journal of Nursing Care Quality	2019	Quality improvement, Before-after empirical study for 18 months	To improve the controlled drug process in the hospital, aiming to reduce nurse journeys to the pharmacy by 25% and release nursing and pharmacy time	Hospital, Lean Six Sigma (LSS)	Lean Six Sigma, with interventions like process redesign, increased frequency of porter-led delivery service, and streamlining checking requirements	Decrease in nurse journeys to the pharmacy for drug collections, time savings for staff, and efficiency of controlled drug process.	A 44% decrease in nurse journeys to the pharmacy for drug collections post-intervention, maintained after 18 months; significant reduction in nursing time spent on controlled drug collections and deliveries, leading to a savings of 661.5 nursing hours over 18 months​	Apply Lean Six Sigma principles to streamline medication management processes in healthcare settings, effectively releasing nursing and pharmacy time for patient care.
Maria Ángeles Castro et al. [68]	Spain	European Journal of Hospital Pharmacy	2019	Empirical study at Hospital de Poniente de Almeria	To identify, prioritize, and map risks in hospital pharmacy processes	Hospital Pharmacy, Healthcare (HFMEA), Risk Management, RPI	Implementation of FMEA methodology to assess and prioritize risks in hospital pharmacy processes. Risk Priority Index (RPI), Criticality Analysis	Identify failure modes, associated adverse events, and causes in various pharmacy areas and processes.	Identification of 99 failure modes associated with 80 adverse events and 129 causes across eight hospital pharmacy areas/subprocesses	Continuously update procedures and use risk maps to identify and prevent risks in hospital pharmacy processes. Emphasize the implementation of safe medication practices and risk management strategies.
Arafeh et al. [69]	Jordan	Journal of Healthcare Engineering	2018	Quality Improvement at King Hussein Cancer Center (KHCC), Amman, Jordan	To reduce patient discharge time in a cancer treatment hospital using the Six Sigma methodology	Hospital Pharmacy, Six Sigma DMAIC, Process Improvement	Patient discharge time is the efficiency of the discharge process. Patient satisfaction, bed availability, operational efficiency	Reduction in patient discharge time	A 54% reduction in patient discharge time from 216 minutes; improved process efficiency and communication; system-wide management improvements	Apply Six Sigma DMAIC and Discrete Event Simulation in healthcare for effective process improvement, focusing on stakeholder engagement for sustainable improvements.
Tadlaoui et al. [70]	Morocco	Journal of Applied Engineering Science	2018	Empirical Study at Sidi Lahcen Hospital, a prefectural hospital center in Skhirat-Temara, for four months (February to April 2016)	To apply Lean Six Sigma in a public hospital to improve pharmaceutical management processes	Hospital Pharmacy, Lean Six Sigma, LSS Optimization	Implementing Lean Six Sigma methodology, process modeling, and performance indicator development. Process efficiency, pharmaceutical management performance. Healthcare service quality, resource optimization	Improvement in pharmaceutical logistics and management processes	Improved process modeling and identification of key performance indicators for pharmaceutical management	Suggest continuing the DMAIC steps to improve further and simulate pharmaceutical management processes
Kieran et al. [71]	Ireland	International Journal for Quality in Health Care	2017	Before-after study [Quality improvement with LSS DMAIC (Define, Measure, Analyse, Improve and Control) approach] at a 20-bed orthopedic ward in a large teaching hospital	To improve efficiency, reduce interruptions, and shorten service time.	Hospital, Lean Six Sigma (LSS), Efficiency, DMAIC	Changes in processes, Flowchart, Pick chart, LSS and DMAIC	Average number of interruptions, average drug round time, and variation in time taken to complete drug round	The average drug round time decreased from 125 min to 51 min 75% reduction in drug supply interruptions. Decrease from 12 to 11 in the average number of interruptions per drug round.	Apply Lean Six Sigma and continuous improvement methodologies for enhancing pharmacy services and patient safety.
Monreal et al. [72]	Spain	Farmacia Hospitalaria	2017	An observational (transversal) and retrospective study in a general hospital of reference with 1,000 beds, 850 with Assisted Electronic Prescribing System (AEPS), with over 1,000 licenses for prescribing physicians, and 1,450 pharmaceutical products available in the system for prescription. The LSS project was conducted between May 2014 and May 2015	To reduce the alert fatigue in Assisted Electronic Prescribing System (AEPS)	Hospital, Lean Six Sigma (LSS), DMAIC Alerts	DMAIC, Alerts generated during two trimesters (before and after the intervention), obtained from the AEP system. For the qualitative analysis of the project, 496 prescriptions in total were reviewed	Number of false-positive alerts in the electronic prescription system	Reduction of 25% in false positive alerts. 4.3 hours per month are saved in prescription and validation time	Use LSS to improve the quantitative and qualitative alert system in an Assisted Electronic Prescription Program and reduce alert fatigue.
Karel et al. [73]	United States of America (USA)	American Journal of Health-System Pharmacy	2017	Quality improvement	To develop a standardized, efficient process for adding and removing medications from the formulary and changing their formulary status.	Hospital Pharmacy, Lean Management, 5S, Process Optimization	Implementation of lean principles like value stream mapping and 5S methods. Creation of standardized templates and shared web-based checklists for formulary status changes.	Streamlined formulary management process with improved coordination and communication, efficient workflow, and enhanced staff satisfaction	More efficient and coordinated formulary management process	Apply performance improvement strategies to nonphysical processes such as formulary management. Implement lean methodology in a coordinated formulary management process, improving resource utilization and patient care.
Shiu and Mysak [74]	Canada	Canadian Journal of Hospital Pharmacy	2017	Empirical study and process improvement project at The University of Alberta Hospital from February to November 2014	To evaluate and improve the efficiency of clinical pharmacist practice in a tertiary care setting through the elimination of non-value-added activities	Hospital Pharmacy, Lean Methodology, Process Improvement, Workflow	Application of Lean principles to identify and eliminate non-value-added activities in clinical pharmacists' workflow, focusing on reducing duplication of work and improving process efficiencies	Reduction in non-value-added activities and improvement in clinical efficiency, measured by changes in time allocation for patient care activities.	Reduction of 31 minutes per day for each pharmacist in non-value-added activities, leading to increased patient assessment time.	Implement standardized work processes for consistent practice and further explore the sustainability and impact of Lean interventions in clinical pharmacy practice.
Goga et al. [75]	United States of America (USA)	The Consultant Pharmacist	2017	Historically controlled study in significant psychiatry care 300 beds hospital from November 2012 to October 2013	To evaluate the effect of applying lean methodology in improving prescribing patterns and reducing inappropriately prescribed antipsychotics with the diagnosis of agitation	Hospital, Lean Methodology, Root Cause Analysis (RCA), Multidisciplinary	The multidisciplinary team used lean methodology to identify the root cause and Interventions necessary to reduce inappropriately prescribed antipsychotics with the diagnosis of agitation	Rate of inappropriately prescribed antipsychotics with diagnosis of agitation	90% reduction of inappropriately prescribed antipsychotics with the diagnosis of agitation	Apply lean methodology to improve prescribing patterns, eliminate waste, and reduce cost
Plas et al. [76]	Netherlands	BMJ Quality Improvement Reports	2017	Quality improvement project. Before and after the study	To reduce parenteral medication administration errors and associated potential risk of harm using Lean Six Sigma strategies	Hospital, Lean Six Sigma (LSS), DMAIC	Lean Six Sigma DMAIC cycle; interventions included substitution of bolus injections with infusions, education, availability of administration information, and drug round tabards	Reduction in medication administration errors and potential risk of harm	Improvement in medication administration safety after the intervention showed that at least one error occurred in 40 (68%) administrations on the intervention ward. However, none of these administrations (0%) were associated with potential risk of harm	Employ Lean Six Sigma methodologies in healthcare settings to systematically reduce medication administration errors and associated risks
Al Kuwaiti [77]	Saudi Arabia	International Journal for Quality Research	2016	A case study conducted in the outpatient pharmacy of the King Fahd Hospital of the University (KFHU) during the year of 2014	To reduce medication errors in the hospital outpatient pharmacy by 20%	Hospital Pharmacy, Six Sigma, DMAIC, Define, Measure, Analyze, Improve, Control	Implement Six Sigma DMAIC methodology, including Define, Measure, Analyze, Improve, and Control stages. Key metrics used included parts per million (PPM) and sigma ratings of various error categories	Reduction in medication errors, improvement in sigma ratings	Prescription/data entry errors reduced from 56000 PPM (sigma rating 3.09) to 5000 PPM (sigma rating 4.08). Improvement in sigma ratings from 3.09 to 4.08	Implement Six Sigma DMAIC methodology to significantly reduce medication errors and enhance patient safety and staff productivity in hospital pharmacy settings​​​
Abuhejleh et al. [78]	United Arab Emirates (UAE)	BMJ Innovations	2016	Case study of a public hospital in the UAE employing Lean management strategies	To investigate the critical success factors for the effective diffusion of Lean innovation in healthcare projects in the UAE	Hospital, Pharmacy, Lean, Management, KPIs, Waiting, Time	Implementation of Lean Six Sigma methodology focusing on improving outpatient pharmacy processes. Key Performance Indicators (KPIs) were based on time reduction and process efficiency	Reduction in outpatient pharmacy waiting time, improved process efficiency, and patient satisfaction	The average waiting time in the outpatient pharmacy project dropped from 45-60 minutes to 4-6 minutes. (90% reduction)	Embrace and adopt Lean management in healthcare projects to significantly improve patient access, reduce waiting times, and foster a culture of innovation and safety.
Shah et al. [79]	United States of America (USA)	Journal of Oncology Practice	2016	Quality Improvement Project at an Academic Medical Center	To examine and improve the safety of oral chemotherapy (OC) prescription processes	Pharmacy, Lean Six Sigma, LSS	An electronic medical system routes OC orders to an oncology-specific outpatient pharmacist for review​​—number of orders reviewed, types of interventions made, and effectiveness.	Effectiveness of pharmacist involvement in reviewing OC orders, types of interventions, and changes in prescription safety.	Over seven months, 63 orders for OC were placed for 45 patients, all reviewed by pharmacists (rate of review 100%), resulting in 22 interventions (35%). Interventions included dosage adjustments, interaction drug identification, and additional monitoring recommendations.	Involve an oncology-trained pharmacist in the review of OC, as it is crucial in OC prescription safety.​​
Furukawa Pde et al. [80]	Brazil	Revista Brasileira de Enfermagem (Brazilian Nursing Magazine)	2016	Before and after study using Lean Six Sigma methodology in a large hospital with 446 beds located in São Paulo between February and September 2010, involving the central pharmacy service and nursing service of a medical-surgical clinic unit	To analyze environmentally sustainable actions in the medication process from prescription reception to waste discard by nursing	Lean Six Sigma, LSS	Implementation of sustainable practices in the medication process, focusing on reducing waste and optimizing resource use	Changes in the amount and type of waste generated, efficiency in waste management.	Pharmacy service saw a 74.8% reduction in chemical, infectious, and sharps waste, a 33.3% increase in common recyclable waste, and a 20% increase in common non-recyclable waste.	Implement sustainable actions to optimize resources, reduce waste, and achieve environmental and cost benefits.
Nabelsi and Gagnon [81]	Canada	International Journal of Production Research	2016	An exploratory case study used an inductive research design and was conducted over one year in two Canadian public hospitals.	To develop an IT strategy for integrating pharmacy and medical equipment supply chains using lean and agile principles.	Hospital Pharmacy, Lean, IT, Strategy	Integration of supply chains through IT strategy, focusing on lean and agile principles for better efficiency and patient care.	Improvement in supply chain efficiency, reduction in costs, and enhancement of patient care quality	Effective hospital supply chain management (SCM) practices enhanced by IT and aligned with lean and agile principles improved supply chain efficiency, reduced costs, and enhanced the quality of patient care.	Integrate hospital pharmacy and medical equipment supply chains using IT and lean agile strategies to improve efficiency, reduce costs, and enhance patient care.
Fisher et al. [82]	United States of America (USA)	BMC Health Services Research	2016	Continuous observation time-motion study in the Birmingham Free Clinic dispensary	To measure how pharmacists allocate their time with the ultimate goal of reducing waste in non-value-added tasks	Pharmacy, Lean Management, Time	Implementation of a time motion study to observe and categorize pharmacists' activities. Task subcategories were developed for the study; inter-rater reliability was measured using Cohen’s kappa.	Allocation of pharmacists' time across various tasks	Four high-level workflow categories occupied almost 95% of pharmacist time: prescription preparation (39.8%), clinician interaction (21.5%), EMR operations (14.8%), and patient interaction (18.7%). Pharmacists invested the most time in prescription preparation, with 21.8% of pharmacist time spent handwriting medication labels. The average value quotient was 40.3%, indicating that pharmacists spend more than half of their time on tasks they consider non-value-added​	Use lean methodology to understand time utilization in pharmacy settings and identify process improvement areas.
Nayar et al. [83]	United States of America (USA)	International Journal of Health Care Quality Assurance	2016	Observational Case study conducted at VHA medical center	To develop recommendations for eliminating wasteful processes and implementing a more efficient and effective process for managing medications	Lean Six Sigma, LSS DMAIC	Application of Lean Six Sigma principles. It involved assessing compliance with policy, collecting data on current processes, and drafting recommendations for improvement.	Improved efficiency and effectiveness in the process	The study identified process bottlenecks that can be targeted for improvement	Apply Lean Six Sigma techniques in healthcare settings to streamline medication management processes to improve efficiency and patient satisfaction.
Lamm et al. [84]	United States of America (USA)	American Journal of Health-System Pharmacy	2015	A three-phase study using Lean Six Sigma methodology	To optimize workflow and efficiency in chemotherapy preparation in an adult infusion clinic	Hospital, Lean Six Sigma, LSS, Kaizen	Implementation of Lean Six Sigma principles	Turnaround Time for Chemotherapy Preparation	Reduced turnaround time from 60 to 26 minutes, representing a 57% decrease	Implement Lean Six Sigma for improved efficiency and workflow
Green et al. [85]	United Kingdom (UK)	International Journal of Pharmacy Practice	2015	Observational study using work-sampling in a district general hospital in the North West of England part of the National Health Service (NHS)	To evaluate the activities of clinical pharmacists concerning the seven wastes described in Lean	Hospital, Clinical Pharmacy, Lean Management, Waste	Waste walk technique to categorize activities into waste and non-waste	Proportion of time spent on waste activities	Of 1440 observations, 342 (23.8%) activities did not add value and were categorized as waste.	Observe the clinical pharmacist’s activities and implement practical steps to ensure their time is used productively and implement lean methodology to reduce waste and improve the efficiency of the clinical pharmacy program outcomes.
Prasetya et al. [86]	Indonesia	International Journal of Pharmaceutical Sciences Review and Research	2015	Case study with Lean Management Approach at Pharmacy department of Santa Maria Hospital. Research begins with observations on April 1 to 30, 2014	To identify the root cause of the problem of waste in the drug procurement process	Hospital Pharmacy, Lean Management, FGD, RCA	Time for activities that do not add value. Mapping process and issues surrounding procurement process. After the mapping process described, focus group discussions (FGD) were conducted to seek waste and identify the cause using the root cause analysis (RCA) 5 whys.	Identification of various forms of waste in the drug procurement process, such as over-processing, overproduction, and issues in inventory, communication, and organization	I found that 60% of the activities did not add value. Increase in process cycle efficiency from 7.68% to 14.42%	Implement Lean methodology to identify and reduce waste in the procurement process.
Arafeh et al. [87]	Jordan	International Journal of Six Sigma and Competitive Advantage	2014	Case study at a local hospital specialized in cancer treatment	To reduce patients' waiting time in a cancer pharmacy using the Six Sigma DMAIC framework	Hospital, Lean Six Sigma (DMAIC), Waiting, Time	Application of Six Sigma methodology; Discrete event simulation (DES) and design of experiments	Reduction of patients' waiting time	Reduction of patients' waiting time by 50%	Encourage the adoption of Six Sigma in healthcare for process improvement
Beard et al. [88]	United Kingdom (UK)	European Journal of Hospital Pharmacy-Science and Practice	2014	Case Study, Change Management Techniques, Improvement projects with Lean Methodologies	To improve services in a hospital pharmacy department in alignment with the NHS Quality Innovation Productivity and Prevention (QIPP) agenda, focusing on increasing service value, reducing waste, increasing efficiency, and improving clinical services without additional staffing.	Hospital Pharmacy, Lean Management, Change	Application of various change management techniques and lean methodologies for service improvement. Prescription turn-around times, delays in chemotherapy provision, rate of inpatient clinical pharmacist consultations, medicines reconciliation rates	Improved prescription turn-around times, reduced chemotherapy delays, increased patient consultations, and improved medicines safety.	Prescription turn-around times reduced to 25 minutes or less, chemotherapy delays decreased from 60% to less than 5%, clinical pharmacists see 98% of inpatients daily, and 92% of patients have their medicines reconciled within 24 hours of admission.	Apply standard change management techniques in hospital pharmacy for significant service improvements. Overcome department size limitations to enhance quality and efficiency in dispensary and chemotherapy units and address patient safety concerns effectively without increasing staff.
Sullivan et al. [89]	United States of America (USA)	American Journal of Health-System Pharmacy	2014	Quality improvement initiative: Time trials, workflow mapping, and impact analysis at the outpatient oncology pharmacy of Yale-New Haven Hospital	To improve workflow and efficiency	Hospital Pharmacy, Lean Methodology Non-value	Lean methodology in oncology pharmacy (workflow optimization, elimination of non-value-added steps)	Efficiency in medication delivery and workflow	Pharmacist and product verification times were reduced by 33% and 52%, respectively, and medication delivery time by 47%, with overall turnaround time shortened by 20 minutes.	Implement Lean methodology to streamline hospital pharmacy operations and enhance efficiency and service quality.
Abdelhadi and Shakoor [90]	Saudi Arabia	Leadership in Health Services	2014	An observational study, direct observation, and data collection in inpatient and outpatient pharmacies at a large regional hospital in Abha, the southern part of the Kingdom of Saudi Arabia, for one week in March 2013	To measure and compare the service quality and efficiency of inpatient and outpatient pharmacies using lean manufacturing principles, specifically focusing on takt (rate) time as a metric	Hospital Pharmacy, Lean Methodology, Efficiency, Takt Time, Cycle Time	Lean manufacturing concepts focused on reducing waste and improving efficiency; the key metric used was takt time. In this case, takt time = available production time per day/ customer demand per day. Also, cycle time used = how long it should take to produce the end product. It includes the value-added and the non-value-added activities	Efficiency of prescription processing in inpatient and outpatient pharmacies	The inpatient pharmacy processed 728 prescriptions with an average time of 3.94 mins per prescription. The outpatient pharmacy handled 992 prescriptions with an average time of 38.28 mins per prescription. The inpatient pharmacy is more efficient due to handling fewer prescriptions or streamlined processes	Apply lean manufacturing principles to hospital pharmacy departments for efficiency improvement
Gijo and Antony [91]	India	Quality and Reliability Engineering International	2014	Case Study using Lean Six Sigma methodology, specifically DMAIC (Define, Measure, Analyze, Improve, Control) at a Super-specialty hospital attached to a manufacturing company with the improvement target set for approximately five months	To reduce patient waiting time in the outpatient department (OPD) of the hospital using Lean Six Sigma strategies	Hospital, Lean Six Sigma, LSS, DMAIC, Waiting, Time	Lean Six Sigma tools, process mapping, cause and effect analysis, software for real-time updates of medicine stock, and process capability analysis. Reduction of patient waiting times as a critical to quality (CTQ) factor, with specific interventions implemented as key process improvements (KPIs)	Reduction in patient waiting times in OPD	A reduction in average waiting time from 57 minutes to 24.5 minutes (57% improvement) and a reduction in standard deviation from 31.15 to 9.27 minutes (70% improvement)	Employ Lean Six Sigma methodology in healthcare to improve service quality.
Breslin et al. [92]	United States of America (USA)	Professional Case Management	2014	Quality improvement project using Lean Six Sigma methodology for improving discharge processes in a healthcare setting at a Private, not-for-profit healthcare system in Atlanta, GA, with two acute care facilities and a long-term acute care hospital	To reduce readmissions for heart failure, acute myocardial infarction, and pneumonia by improving the discharge process and transitions of care from hospital to post-acute care using Lean Six Sigma methodology	Hospital, Lean Six Sigma, LSS	Implementation of Lean Six Sigma to streamline discharge processes. Implementing a redesigned discharge process included establishing a post-discharge clinic and improving coordination of care. Focus on reducing readmission rates, improving patient satisfaction with the discharge process, and providing effective post-discharge follow-up.	Reduction in readmission rates, improvement in patient satisfaction regarding discharge information	In the first 30 days, the readmission rate for patients seen in the clinic was 6.5% compared to the baseline rate of 16.2%. Patient satisfaction with discharge information increased to over 90% in the second quarter of 2013 from 76% in the last quarter of 2012	Implement comprehensive discharge processes to prevent patient harm and improve outcomes; leverage Lean Six Sigma tools to structure quality improvement projects.
Baril et al. [93]	Canada	Journal of Medical Systems	2014	Case study, direct observation, time study, work sampling, incident/accident reporting at Health and social services center (HSSC) in Québec, which includes a hospital and six nursing homes	To assess the impact of medication distribution technology on the performance of a pharmacy within a HSSC, the performance of a care unit in a nursing home, and medication-use process safety	Hospital Pharmacy, Lean Approach, Performance	Implementation of an automated packaging device and mobile medication dispensing carts; Lean approach for performance assessment	Time to prepare medicines. Time to distribute medicines. Pharmacy performance, care unit performance, medication-use process safety	Reduction of 92% in time to prepare and a reduction of 6% in time to distribute medicines	Continue optimizing medication distribution processes using technology and Lean principles to enhance efficiency and safety.
Villafranca et al. [94]	Spain	American Journal of Health-System Pharmacy	2014	The study used FMEA to analyze the preparation and dispensing of neonatal parenteral nutrition in a hospital pharmacy service at a general hospital.	To identify potential errors and to enable the implementation of measures to improve the safety of neonatal parenteral nutrition (PN)	Hospital Pharmacy, (FMEA), Risk Management, RPI	FMEA was used as a quality management tool to assess risks in the neonatal parenteral nutrition process, and a checklist was developed to reduce errors. The risk Priority Index (RPI) was a key performance indicator.	Reduction in medication errors and improvement in the safety of the neonatal parenteral nutrition process	A reduction of the mean RPI from 137 to 48 after implementing a checklist	Adopt FMEA and similar proactive risk assessment tools to detect and mitigate potential errors in medication management processes, especially in high-risk areas such as neonatal care
Arias Rico and Jagwani [95]	Spain	European Journal of Hospital Pharmacy	2013	Application of lean methods to pharmacy compounding services, with data collected from workflow assessments and value stream mapping at Hospital Universitario Doctor Negrin in Las Palmas, Gran Canaria, from January 2010 to December 2011	To improve the efficiency of compounding services in a hospital pharmacy by implementing lean methods	Hospital Pharmacy, Lean Approach, VSM, Quality	Value Stream Mapping (VSM), lean techniques for process improvement, and quality assurance strategies	Efficiency and quality of pharmacy compounding services	Significant improvement in the quality (First Time Quality improved from 56% to 95%) and efficiency of compounding services, with a noted increase in compounding sterile preparation activities post-implementation (94.4% increase)	Implement lean methods to streamline healthcare processes, engage front-line staff in problem identification and solution development, and continuously assess and adapt processes for sustained improvement
Lorimer et al. [96]	Canada	Canadian Journal of Hospital Pharmacy	2013	Case study as practice model change in a tertiary care teaching hospital, focusing on transitioning toward a patient-centered, integrated pharmacy practice model at Royal Jubilee Hospital, a 453-bed tertiary care teaching hospital part of the Vancouver Island Health Authority. Ongoing change started in 2008 and continued through 2012	To redesign the pharmacy practice model to enhance patient care by integrating pharmacists into ward areas and shifting the focus from drug distribution to patient-centered care.	Hospital Pharmacy, Clinical Pharmacy, Lean Methodology, Leadership	Introduction of a new pharmacy leadership team, creation of a troubleshooter pharmacist role, re-orientation of pharmacists toward clinical practice, implementation of a lean design project	Increase in ward areas covered by clinical pharmacists, percentage of clinical coverage throughout the hospital, and reallocation of pharmacist resources from dispensary to wards	Increase in ward areas with clinical coverage from 20% in 2008 to 100% in 2011	Develop a formal role for clinical pharmacists while providing high-quality pharmacy services seven days per week​​.
Lingaratnam et al. [97]	Australia	Journal of Oncology Practice	2013	Service improvement project Lean improvement methodologies, process mapping, staff and patient tracking, opinion surveys, medical record audits at Peter MacCallum Cancer Centre, East Melbourne, Victoria, Australia. The project spanned ten months with continuous engagement	To reduce patient wait time and improve equity of access to the chemotherapy day unit (CDU)	Hospital Pharmacy, Lean, Leadership, Waiting, Time	Lean methodology, performance data suite development, process optimization. Reduction in median patient wait times, improvement in treatment commencement times	Wait times, start of treatment, chemotherapy wastage, system efficiency	Waiting time for the patient was reduced by 38% Time for releasing the first dose reduction of 28% Medicine loss by expiry or reworking reduction of 76%	Use lean methodology to understand and manage complex system constraints, which can result in improved treatment access and reduced waiting times
Jenkins and Eckel [98]	United States of America (USA)	American Journal of Health-System Pharmacy	2012	Observational study with workflow analysis	To report the results of a workflow analysis at a large central outpatient pharmacy and explore potential efficiencies attainable through workflow enhancements​​	Hospital Pharmacy, Lean	Two models: Model A expanded the standard duties of pharmacy technicians. Model B offered an even more significant reduction in non-value-added activities by changing workflow and technician responsibilities​​. Efficiency in pharmacist time use, reduction in non-value-added activities	Shift in pharmacist activities from non-value-added to value-added activities, such as patient engagement, order verification, and counseling.	Model B showed a 74% potential reduction in non-value-added activities	Optimize the use of value-added pharmacist time in the dispensing process by reorganizing workflow and reallocating tasks
Bruchet et al. [99]	Canada	Canadian Journal of Hospital Pharmacy	2011	Descriptive Study on general practices in clinical pharmacy	The aim is to improve the quality of clinical pharmacy services by identifying and implementing high-value (Quality Actions)	Pharmacy Services, Clinical Pharmacy, Quality Actions	The strategy involves defining and capturing actions necessary to achieve a standard of care for specific conditions, termed “Quality Actions.”	The measurement involves documenting whether a quality action has been considered or performed and by whom	Structured methodologies like Lean enhanced operational efficiency	Integrate quality actions into daily clinical pharmacy practice to provide evidence-based, safe, effective, and efficient drug therapy
Newell et al. [13]	United States of America (USA)	Journal for Healthcare Quality	2011	Service improvement project. Before-after study. Application of Toyota Production System (TPS) techniques to medication delivery in two adult care hospitals within the Spectrum Health Regional Health Care System	To improve medication safety and reduce the time needed for nurses to retrieve patient medications	Hospital Pharmacy, Lean, Toyota Production System (TPS), KANBAN, PDCA, Pareto, Waiting, Time	5S (focus on workplace design during simulation phase); KANBAN; PDCA and Pareto diagram	Delivery process improvements, patient safety and satisfaction, nursing satisfaction, and pharmacy efficiency	Medication returns decreased by 60% on the Neurosciences (four south) pilot unit and 30% on the Medical-Surgical (six south) pilot unit. Reduced returns have generated several benefits, including. (a) decreased nursing processing time, (b) decreased pharmacy technician processing time, Moreover, (c) reduced medication waste. Increase of 29% in nursing team’s satisfaction. Reduction 2.5 h/day in the time spent by the nursing staff to locate medications. - Reduction in the nursing waiting time to receive medications from the pharmacy (5 H/day) resulting from the optimization of the Pharmacy medication the delivery process to the nursing units	Encourages the adoption of TPS techniques in healthcare settings to improve processes and outcomes
Al-Araidah et al. [100]	United States of America (USA)	Journal for Healthcare Quality	2010	Case Study/Quality Improvement Project at a local hospital	To reduce lead time in the drug dispensing process at an inpatient pharmacy	Hospital Pharmacy, Lean Six Sigma (LSS) Methodology, DMAIC, Utilization	Application of Lean Six Sigma tools to streamline the drug dispensing process, eliminating unnecessary steps and optimizing resource utilization	Reduction in drug dispensing cycle time and improvement in process efficiency	45% reduction in the drug dispensing cycle time	Implement Lean Six Sigma tools in healthcare settings to improve efficiency and reduce lead times, particularly in pharmacy operations.
Aboumatar et al. [101]	United States of America (USA)	The Joint Commission Journal on Quality and Patient Safety	2010	Quality improvement project. Application of Lean Sigma methodology, including Failure Mode and Effects Analysis (FMEA) and process redesign at The Weinberg pharmacy, Sidney Kimmel Cancer Center, The Johns Hopkins Hospital	To safeguard the chemotherapy preparation process against errors and increase compliance with USP 797 regulations	Lean, Sigma, Mistake-proofing	The article describes interventions such as workspace redesign, process redesign, and the development of standard operating procedures for pharmacy staff to mistake-proof the chemotherapy preparation process.	The reported medication errors reaching patients and the reported near misses	Reduction in the mean time to prepare chemotherapeutic agents by 9%, significantly reducing distractions for pharmacists. Reported medication errors reaching patients and requiring monitoring decreased, whereas reported near misses increased.	Implement Lean Sigma solutions to enhance chemotherapy preparation safety and efficiency. Develop awareness of patient safety and create a dynamic risk map to identify errors before reaching patients. Expand mistake-proofing interventions to various pharmacy areas.
Kumar and Kwong [102]	United States of America (USA)	Technology and Health Care	2010	The experimental case study utilized Six Sigma tools such as Service Blueprinting, Cause-effect diagram, Gap Analysis, and Poka-Yoke measures.	To integrate internal operations of a pharmacy using Six Sigma tools and improve efficiency	Pharmacy, Six Sigma, Poka-Yoke, Mistake-Proofing, RCA, Cause-effect	Use of Six Sigma methodology, including service blueprinting, gap analysis, and mistake-proofing measures for process improvements	Improvement in pharmacy process flow and efficiency, identification of critical areas for enhancing customer and employee satisfaction	Findings indicate that the Six Sigma tools are very applicable and quite effective in helping to streamline and integrate the pharmacy process flow	Implement Six Sigma tools in retail pharmacy operations to enhance process efficiency, improve customer and employee satisfaction, and streamline workflow
Beard and Wood [103]	United Kingdom (UK)	The Pharmaceutical Journal	2010	Analysis of current pharmacy dispensing processes at Musgrove Park Hospital, part of the Taunton and Somerset NHS Foundation Trust	To reduce average inpatient prescription dispensing times to less than one hour without increasing staffing levels or adversely affecting patient safety	Hospital Pharmacy, Lean Principles, FMEA, Processes	Process and value mapping, capacity estimation, failure mode effect analysis of current processes	Dispensing times, staff satisfaction	Median dispensing times decreased from 188 minutes to 27 minutes. The standard deviation was 7 minutes, and 65% of daily work was completed before 1 pm. Dispensary closed at 5.30 pm consistently, and dispensing error rates remained unchanged.	Applying interventions that reduce wasteful activities and increase workflow efficiency can consistently reduce dispensing times without increasing staff or adversely affecting patient safety.
Hintzen et al. [104]	United States of America (USA)	American Journal of Health-System Pharmacy	2009	Quality improvement project at University of Minnesota Medical Center, Fairview	To improve workflow, reduce waste, and achieve substantial cost savings in the inpatient pharmacy's sterile products area (SPA) and inventory area. Relevant Key Terms: Lean process improvement, 5S, value-stream mapping, waste reduction, cost savings	Hospital Pharmacy, Lean Methodology, 5S, VSM, Waste	Implementation of Lean techniques such as 5S and value-stream mapping in inpatient pharmacy. Reduction of missing doses, errors, patient-specific waste, and improvement of workflow and operational efficiency	Cost savings, waste reduction, workflow improvement, Decreased staffing requirements	SPA waste reduction yielded an annual saving of $275,500. There was an 83% decrease in production errors, and the average number of missing doses was reduced from 53 to 13.8 per day. Medication inventory decreased by $50,000 and outdated products by 20%.	Implement lean methodology in pharmacy settings to achieve significant savings and efficiency improvements.
Davis [105]	United States of America (USA)	Hospital Pharmacy	2009	Quality Improvement Project, Examination of the effects of a 5-batch-per-day compounding schedule on waste reduction in sterile product compounding at Exempla Lutheran Medical Center, a 400-bed community nonprofit hospital	To standardize delivery locations and improve just-in-time delivery for sterile medication products, aiming to transform the sterile medication preparation process from a 2-batch to a 5-batch-per-day system	Hospital Pharmacy, Lean Methodology, Mistake-Proofing, Compounding	Lean production concepts, including the implementation of a 5-batch-per-day schedule for sterile product compounding	Reduction in rework and waste in the compounding process	A 64% reduction in rework and waste was achieved by increasing the frequency of sterile product batches from 2 to 5 per day	Investigate the impact of mistake-proofing interventions on medication error rates and adverse events and implement interventions across pharmacies to optimize physical space, minimize visual clutter, streamline preparation and labeling processes, and develop operational management systems that prevent standard error modes

Risk of Bias in the Included Studies

**Figure 2:**
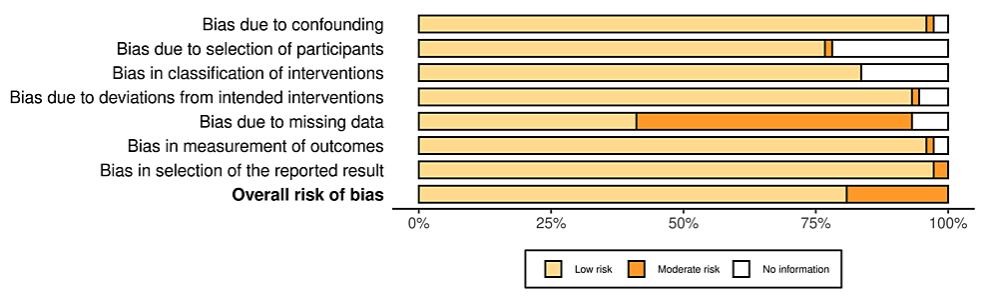
Publication quality risk-of-bias assessment Risk-of-bias VISualization (robvis) used to create risk-of-bias plot [30]

The overall biases resulting from confounding, participant selection, intervention classification, deviation from intended interventions, outcome measurement, and reported results are considered to have a low to moderate risk, with a few studies lacking sufficient information to determine the risk level. Missing data are notable for having a moderate risk. This underscores the need for careful consideration when interpreting the systematic review's findings [29,30]. The evaluation of bias risk in the included studies is presented in the provided bar chart in Figure 2.

Annual Trend of Published Studies from 2009 to 2023

**Figure 3:**
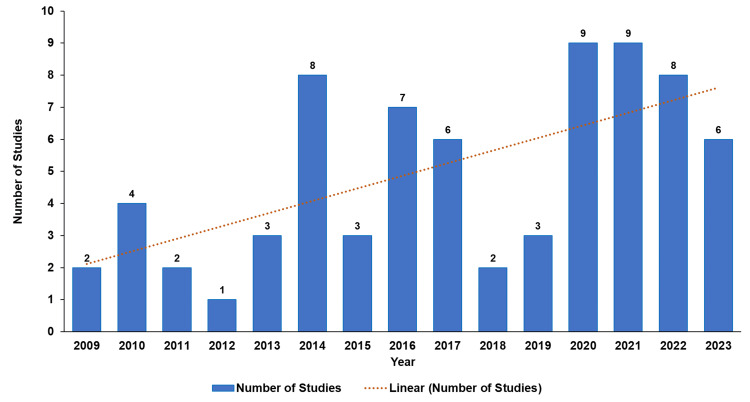
Column chart for the annual trend of published studies from 2009 to 2023‎

Initially, only two studies were published in 2009; However, the volume of papers showed an irregular yet predominantly upward trend throughout the years. Notably, there was a significant surge in 2020, 2021, and 2022, with the number of studies peaking at 9, 9, and 8, respectively. This increase in published studies during these years could indicate a heightened research activity in response to the global COVID-19 pandemic. The increased focus on LSS methodologies is likely a result of the urgent necessity to reduce medication wastage and optimize healthcare processes during that critical period. The above underscored the crucial role of such studies in adapting to the challenges presented by the pandemic, which may have acted as a catalyst for research and innovation in this field worldwide [106]. Figure 3 illustrates the yearly progression of research papers identified through a review search strategy over fifteen years, from 2009 to 2023.

Global Geographical Distribution of Studies

**Figure 4:**
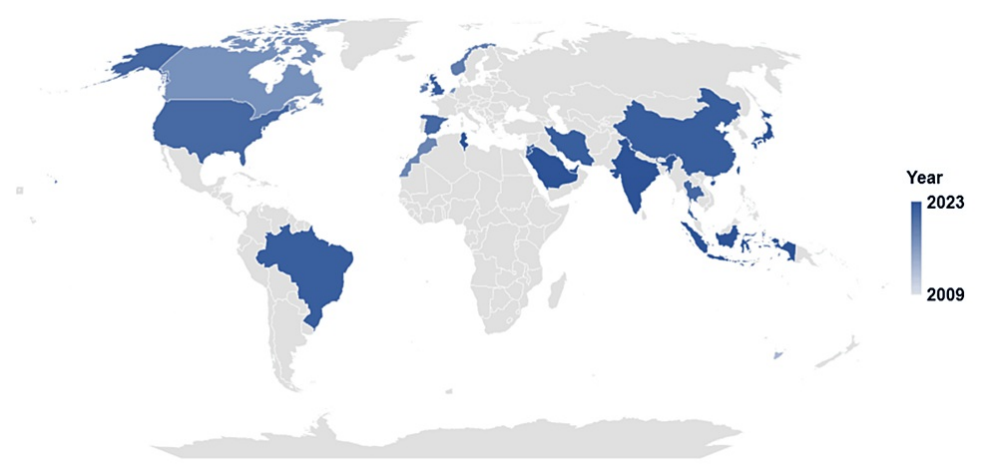
Global geographical distribution of pharmacy Lean Six Sigma studies‎: 2009-2023‎ Map generated using the “Insert Map Chart” option in Microsoft Excel 2021 [33]

**Figure 5:**
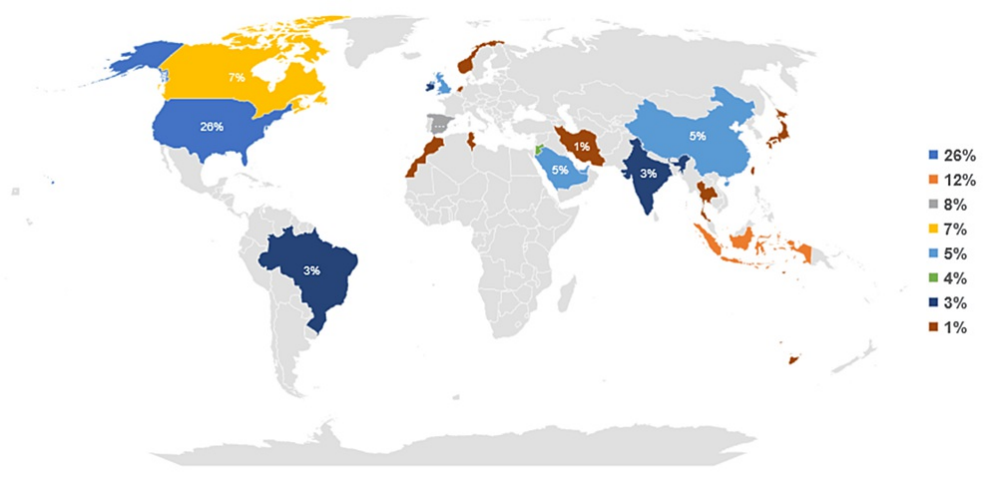
Percentage of global geographical distribution of pharmacy Lean Six Sigma studies Map generated using the “Insert Map Chart” option in Microsoft Excel 2021 [33]

The global geographical distribution of Pharmacy LSS studies from 2009 to 2023 and the percentages across different countries are illustrated in Figures 4, 5, respectively.

Darker shades on the map in Figure 4 indicate countries with a higher frequency of studies. Figure 5 reveals that the United States of America (USA), with 26% of studies, was the predominant contributor during the study timeframe. Alongside the USA, Indonesia (12%), Spain (8%), Canada (7%), China, Saudi Arabia, the United Arab Emirates (UAE), and the United Kingdom (UK) were also projecting in terms of research output with (5%) each. The geographical spread of the studies across different continents highlighted the global interest and the diverse investment in the research areas covered. The research was not confined to any particular region but a worldwide effort with active involvement from multiple countries. Representing countries from continents and areas such as North America, Asia, the Middle East, Europe, and Australia painted the extensive range and critical importance of the subjects investigated in these studies.

Journal Fields

**Figure 6:**
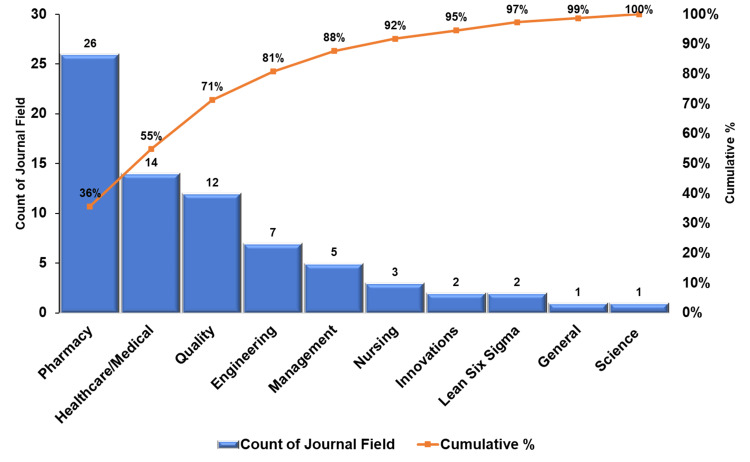
Pareto chart showing fields‎ of the included journals

The Pareto chart in Figure 6 exhibits the distribution of published research in different disciplines of distinct journals. It clearly pointed out a notable dominance of 26 journals in the pharmacy sector. The healthcare/medical category ranked second with 14 publications, closely followed by the quality category with 12 articles. Engineering journals were represented with seven articles. This distribution underscored the focus of this review, stressing advancing knowledge and practical application in pharmacy, healthcare, and quality.

Designs of the Included Studies

After a comprehensive analysis of 73 studies conducted in the hospital pharmacy within the healthcare industry, it became apparent that there was a notable emphasis on utilizing practical and observational approaches. Observational studies, in particular, made up a significant portion of the research designs identified. These studies encompassed a wide range of topics, from analyzing the systems within inpatient medication dispensing units to examining the risk management practices in hospital pharmacies. This demonstrated a focus on current practices and their outcomes. Another standard research design was the retrospective descriptive study, which provided valuable insights into past performance and trends over an extended 19-month period [36]. Quality improvement initiatives were also well-represented in the research landscape. These initiatives often utilized the Six Sigma approach and failure mode and effects analysis (FMEA). They frequently involved staff surveys and interventions to improve operational efficiency and patient care. Many studies also employed lean management techniques, indicating a noticeable shift within the healthcare industry toward more streamlined and patient-centric service delivery.

Additionally, case studies played a vital role in the research conducted, as they provided a detailed examination of specific interventions or changes within a single entity, such as a hospital unit or outpatient pharmacy [14]. This approach was crucial for understanding the practical application of theoretical models. While less common, experimental designs also contributed to the empirical evidence base, particularly in process improvement and technology integration. These studies varied in duration, some lasting only a few months while others spanned several years. This highlighted the diverse scopes and scales of research within healthcare improvement initiatives. The methodologies utilized across these studies demonstrated a strong and dynamic approach to understanding and improving healthcare processes, with a clear preference for observational and quality improvement research in real-world clinical settings.

Visualization of Relevant Key Terms

**Figure 7:**
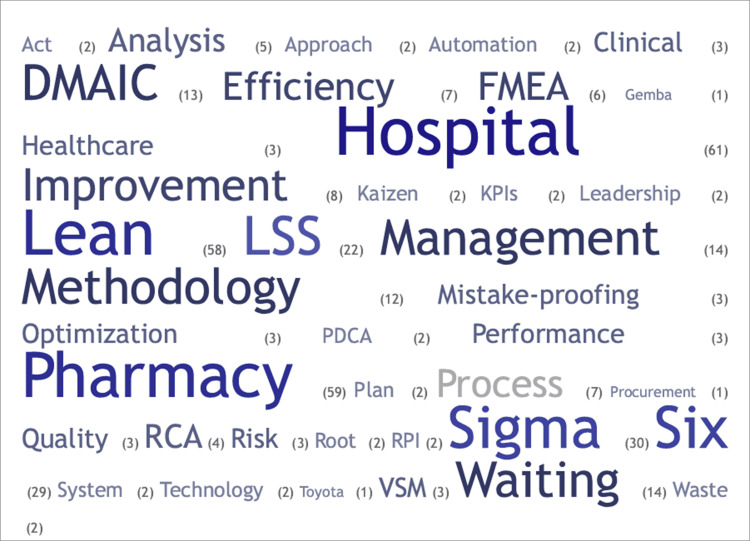
Main keywords visualized as a word cloud The word cloud visualization was obtained from TagCrowd: a web-based word cloud generator [107] Numbers next to each word show the keyword count frequency in the selected articles Credit: Dr. Mohammed Sallam

A word cloud was generated by the author (Figure 7) using a web-based word cloud generator [107] to showcase and highlight the primary themes and methodologies used in the selected articles. This visualization, emphasized the terms hospital, pharmacy, lean, six, and Sigma as the most frequently mentioned terms, indicating that the review focused on LSS practices in the field. The abbreviation LSS, which stands for LSS, was also prominently featured, further highlighting its importance. Moreover, the concepts of improvement, process, management, methodology, and efficiency were frequently referenced, demonstrating the continuous venture of excellence in operational practices in hospital pharmacies. The terms FMEA and define, measure, analyze, improve, control (DMAIC) also indicated a systematic and analytical approach to quality and risk management. While less common, terms like clinical, quality, risk, performance, and healthcare were used in conjunction with other terms to provide a comprehensive overview of the strategic priorities in hospital pharmacy research during the reviewed period.

LSS Tools and Techniques Used in the Context of Hospital Pharmacy

**Figure 8:**
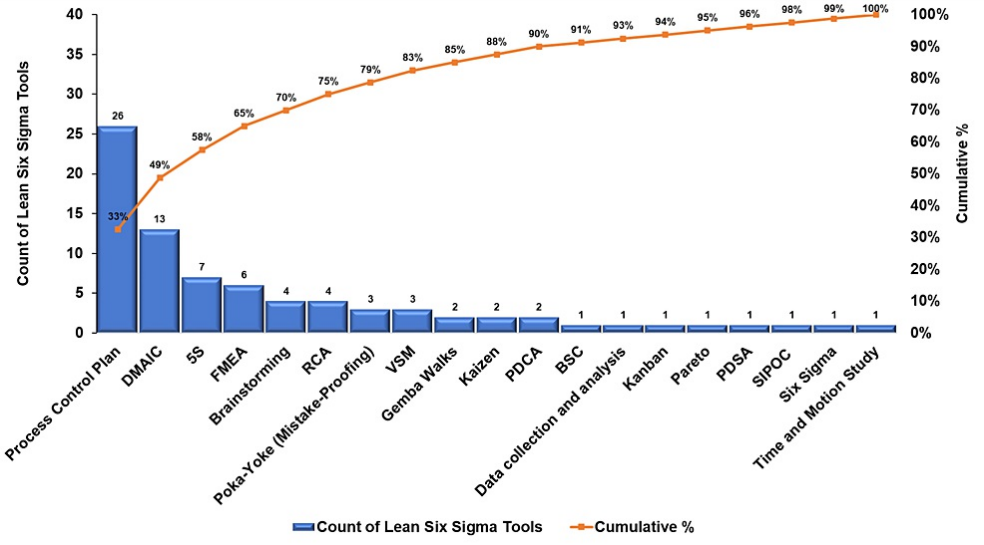
Pareto chart displaying Lean Six Sigma tools and techniques used in the context of hospital pharmacy DMAIC: Define, measure, analyze, improve, and control; 5S: Sort (Seiri), set in order (seiton), Shine (Seiso), standardize (Seiketsu), and sustain (shitsuke); FMEA: Failure mode and effect analysis; RCA: Root cause analysis; VSM: Value stream mapping; Gemba Walk: Japanese term meaning the physical workplace; Kaizen: Japanese term meaning continuous improvement; PDCA: Plan-do-check-act; BSC: Balanced scorecard; Kanban: Japanese term meaning a visual work management system card; Pareto: (also known as the 80/20 rule) is a phenomenon states that roughly 80% of outcomes come from 20% of causes; PDSA: Plan-do-study-act; SIPOC: Suppliers, inputs, processes, outputs, and customers

The Pareto chart in Figure 8 displays the frequency of using different LSS tools and techniques in hospital pharmacy settings. This chart offers considerable knowledge and a detailed guide for industry professionals, enabling them to accurately recognize the most frequently used methods and approaches. By examining the distribution of occurrences illustrated in this visual representation, professionals working in hospital pharmacies can make well-informed choices, prioritize their actions, and effectively allocate resources to achieve the best results in enhancing quality and streamlining processes.

The process control plan was the most widely utilized tool, with a count of 26, indicating its crucial role in managing and controlling processes. Following closely was DMAIC, with a count of 13, showing its significant application in improving processes within the sector. The 5S method ranked third in popularity, with a count of seven, highlighting its implementation for organizing and improving efficiency [108]. Tools like FMEA and RCA, which involve risk assessment and problem-solving, had moderate usage with counts of six and four, respectively. Poka-Yoke (mistake-proofing) and value stream mapping, each with a count of three, were also notable as they are crucial in reducing errors and optimizing workflows. Other LSS tools such as brainstorming, Gemba walks, Kaizen, and PDCA had lower frequencies, ranging between one and two counts, suggesting that they are utilized in more targeted situations or as complements to frequently used techniques. Suppliers, inputs, processes, outputs, and customers, Six Sigma as a standalone tool, and time and motion study each have a count of one, indicating that they have niche or particular use cases in the presented context. The cumulative percentage line graph showed a steady increase towards 100%, demonstrating the widespread adoption of these LSS tools in hospital pharmacies. This upward trend reflected the comprehensive range of methodologies used for optimizing processes, improving quality, and enhancing efficiency in healthcare. The data highlighted the diverse array of LSS terms and methodologies implemented in practice, with some playing a more central role in operations than others [109].

Various Management Strategies

The most commonly reported strategies were quality improvement initiatives and workflow optimizations, illustrated with 22 (30.14%) studies each. Close behind were technological integrations, with 15 (20.55%) studies. On the other hand, cost management, patient-centered care, and staffing models had three (4.11%) studies each. Inventory management enhancements showed two (2.74%) studies. Finally, continuing education, interdisciplinary collaboration, and policy changes were represented by one (1.37%) study for each.

**Figure 9:**
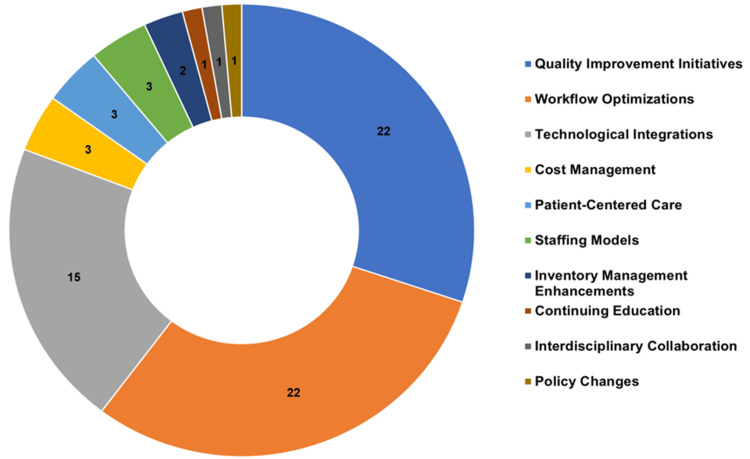
Doughnut ‎chart for different management strategies

Figure 9 presents a doughnut graph that provides objective quantitative data on the frequency of different management strategies utilized. This graph highlighted an apparent inclination toward strategies primarily concentrating on quality improvement, workflow optimization, and technology integration.

Outcome ‎Measured/Analysis

The compilation of studies encompassed a variety of outcomes, highlighting significant advancements and areas of focus in the pharmacy operations management field. Many studies prioritized reducing medication and dispensing errors through strategies like analyzing failure modes in medication management and implementing robust systems, aligning to improve patient safety and care quality. A central theme was the enhancement of workflow efficiencies by reducing disruptions from routine inquiries and improving the speed and dependability of medication dispensing, especially in emergencies. Regarding inventory management, several studies focused on optimizing stock utilization and turnover, notably reducing unnecessary drug usage. The patient-centric approach explored satisfaction levels, wait times, and clinical intervention effectiveness. Operational and financial efficiency examined initiatives' impact on hospitals' financial performance, balancing cost-effectiveness and quality care. Additional research is imperative to analyze the effects of various indicators on hospitals' financial performance. The aim is to attain a balanced state of optimal cost efficiency and exceptional quality of patient care [110]. Staff efficiency and satisfaction involved evaluating processes to enhance working conditions, thereby improving pharmacy services' operational aspects and contributing to higher morale. Quality and safety in medication management were at the forefront of the studies that assessed pharmacy systems' effectiveness in mistake reduction and safety practices. Other studies measured advancing knowledge and skills, especially in lean management principles, reflecting the evolving nature of pharmacy practice. Though not quantified here, interdisciplinary collaboration and sustainable practices in pharmacy operations appeared as themes emphasizing hospital efficiency, patient care pathways, responsible waste management, and environmentally sustainable methods. These studies illustrated a changing landscape in hospital pharmacy management, focusing on safety, efficiency, patient-centered care, and continuous improvement.

Discussion

The systematic review, consisting of 73 studies that examined the implementation of Lean and Six Sigma methodologies in hospital pharmacy operations, yielded 11 valuable insights. Analysis of the results offered a comprehensive understanding of how these methodologies impact various aspects of pharmacy operations. Notably, 26% of the studies highlighted a significant decrease in medication turnaround time. This finding holds great importance given the time-sensitive nature of medication delivery in healthcare settings. The effectiveness of Lean and Six Sigma methodologies in streamlining pharmacy operations was clearly evident and suggested improved operational efficiency in delivering patient care. Process efficiency improvements were a prominent focus in 15% of the studies. These improvements were critical in optimizing pharmacy workflow, resulting in more efficient operations. The emphasis on efficiency indicated that these methodologies have the ability to refine processes for enhanced performance.

Further, 11% of the research emphasized identifying bottlenecks in processes and failure modes and minimizing non-value-added activities. This particular theme closely aligns with LSS's fundamental principles, which greatly emphasize reducing waste and optimizing processes. Another group of studies, also at 11%, focused on enhancing inventory management. This aspect holds considerable significance in hospital pharmacies, as efficient management of pharmaceutical stocks can significantly impact costs and patient care. The studies found that reducing medication errors, which accounted for 9% of the research, was evidence of the improved accuracy and safety inherent in the streamlined processes. This finding is significant in healthcare settings, where errors can have severe and, at times, fatal consequences.

In addition, 8% of the studies found that implementing these methodologies increased satisfaction rates among staff and patients. This suggests that applying LSS tools not only improved operations but also positively impacted overall satisfaction. Another 8% of studies showed reduced medication expenditure, demonstrating the financial benefits of effectively managing resources through these tools. Approximately 6% of the studies indicated improvements in clinical pharmacy activities, showing a shift towards a patient-centered approach and optimized clinical outcomes. Additionally, 3% of the studies highlighted improvements in prescription checking and intervention success rates, highlighting the role of these methodologies in enhancing the quality of pharmacy services. A decrease in workflow interruptions was observed in 2% of the studies, indicating a more consistent and uninterrupted pharmacy service. Lastly, 1% of the studies reported increased knowledge, which is crucial for continuous improvement and adaptation in dynamic healthcare environments.

**Figure 10:**
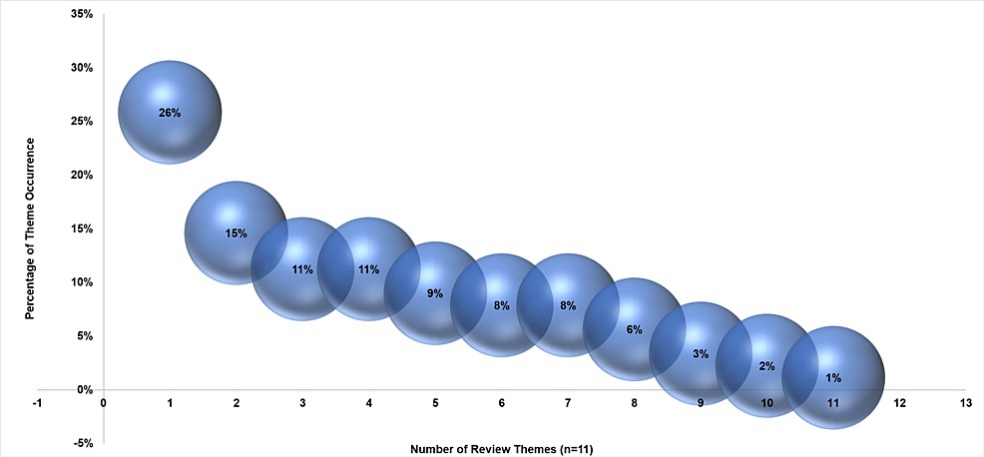
Percentage distribution of theme occurrence in the research Reduction in medication turnaround time (26%), improvements in process efficiency (15%), identification of process bottlenecks, failure modes, and reduction of non-value-added activities (11%), improvements in inventory management (11%), reduction in medication errors (9%), increase in satisfaction rate (8%), reduction in medication expenditure (8%), improvements in clinical pharmacy activities (6%), improvements in prescription checking and intervention success rates (3%), reduction in workflow interruptions (2%), increase in knowledge (1%)

Figure 10 illustrates the percentages of theme occurrence in the review studies.

The structured review and analysis brought to light a piece of persuasive and concrete objective evidence that implementing Lean and Six Sigma methodologies yielded multiple enhancements in hospital pharmacy operations. These improvements encompassed increased efficiency and precision, financial management, and enhanced patient care, highlighting LSS methodologies' extensive and varied influence on pharmacy operations.

This review focused on the range of tools and management approaches employed in hospital pharmacies within the operations management scope under Lean and Six Sigma frameworks [111]. The pharmacy operational performance across the studies strongly correlates with implementing lean healthcare practices [15]. Most of LSS's tools rely heavily on data to comprehend issues [112]. Data is a vital requirement to commence any process enhancement. The incorporation of intelligent technologies enables clients, suppliers, and employees to participate actively in real-time data collection. Implementing LSS in healthcare domains emphasizes the necessity to establish a standardized framework for its prosperous execution across the entire sector [113].

One such tool is the voice of the customer (VOC), which aids in understanding customer needs. Some of the included studies focused on how qualitative data gathered from the VOC was translated into innovations and enhancements in pharmacy services. Additionally, key performance indicators (KPIs) were examined to evaluate the effectiveness of pharmacy processes and measure progress. Examples of pharmacy KPIs include the number of prescriptions processed, the number of errors detected before reaching the patient, the cost per prescription, and wait times. The third tool, critical-to-quality (CTQ), was explored concerning creating and optimizing processes within the pharmacy. The CTQ tree has been introduced within studies, outlining different significance levels. Last, some articles introduced critical success factors (CSFs) as broader goals that pharmacy teams strived for. It emphasized aligning daily activities with vital strategic areas [114].

Key review themes for Lean and Six Sigma-driven transformations in hospital pharmacy operations

Reduction in Medication Turnaround Time and Impact on Patient Experience

Reducing medication turnaround time has been crucial in improving hospital pharmacy services. The review has presented compelling evidence, with various studies documenting significant reductions in turnaround time ranging from 9% to 93%. These notable decreases illustrated a noteworthy improvement in how medications were dispensed, highlighting a broader commitment to enhancing operational efficiency and patient satisfaction. The aggregated data demonstrated a concerted effort within the field to optimize pharmacy workflows, resulting in reduced wait times for patients and improved timely administration of medications. The recurring presence of this theme in multiple studies reaffirms the critical nature of turnaround time in healthcare provision. The progress made in this area is supported by a substantial number of studies, reflecting a collective effort toward more efficient pharmacy practices [39,41,42 48 51,53-55,62,67,69,71,84,87-91,93,97,100,101,103]. Reduction in patient waiting times is an essential goal for healthcare facilities to enhance the overall patient experience. By adopting efficient strategies, pharmacy healthcare providers have effectively reduced waiting times in both outpatient and inpatient settings. This reduction significantly benefited patients, ensuring their valuable time was not wasted, and their healthcare needs were promptly met.

Improvements in Process Efficiency

A central theme from the comprehensive review was the significant improvement in operational efficiency within hospital pharmacies. Several studies [60,61,64,74,86,95] provided quantitative evidence of this enhancement, showing notable efficiency increases ranging from 7% to 88%. These advancements signify a shift in focus toward optimizing pharmacy operations by efficiently utilizing resources and streamlining processes for better productivity. The absence of specific percentages in other studies [45,63,65,66,70,73,102] does not undermine the importance of their contributions to this theme. These studies highlighted the continuous efforts to refine pharmacy workflows, minimize redundancy, and improve service delivery. The results of these improvements were evident in the substantial efficiency gains, showcasing the success of different strategies implemented in various settings.

This prevalent theme emphasizes the ongoing commitment in the field to evolve and adapt to the complex demands of healthcare. By embracing innovative approaches and continuous improvement methodologies, hospital pharmacies can significantly enhance the quality of their services, ultimately benefiting patient care.

Identification of Process Bottlenecks, Failure Modes, and Reduction of Non-Value-Added Activities

The systematic review highlighted another central theme in hospital pharmacy practice. Various studies [47,74,82,85,86,94,98] offered quantitative evidence for this trend, showcasing improvements in efficiency metrics ranging from 7% to 74% by reducing non-value-added activities. These numbers illustrate a substantial advancement in pharmacy operations, emphasizing a deliberate effort to eliminate inefficiencies and optimize workflow. Even in studies where specific percentages were not provided [35,68,83], the recurrence of this theme highlights its significance in today's healthcare landscape. These studies collectively indicated an industry-wide initiative to refine processes, a necessary step for the sustainable provision of healthcare services. Notably, one study [94] reported a 65% reduction in the mean risk priority number, demonstrating the depth of this endeavor. This reduction significantly improved identifying and addressing potential failure points in pharmacy operations, ultimately enhancing patient safety standards. This process involved comprehensive assessments of medication processes, identifying potential areas of improvement, and implementing robust strategies to mitigate errors and enhance overall medication management standards.

These insights portray a proactive approach to continuous process improvement. By identifying and resolving bottlenecks, failure modes, and non-value-added activities, hospital pharmacies are improving their operational efficiency and making notable contributions to the broader goals of healthcare quality and patient safety.

Improvements in Inventory and Supply Chain Management

The systematic review has revealed that improving inventory management within hospital pharmacies has been a significant area of focus. Studies that specifically mentioned quantitative enhancements [52,58,97,104] demonstrated notable progress, with efficiency gains ranging from 20% to 76%. These improvements signify a considerable shift toward more precise and effective pharmaceutical stock management, an essential element of pharmacy operations. Even in studies that did not provide specific percentages [37-39,43,59,81], the recurring emphasis on improvements in inventory management still provides valuable insights. It suggests a widespread recognition of the necessity for and implementation of better inventory control measures. These measures likely include technological advancements for tracking and forecasting, optimized stock rotation, and strategies to reduce waste effectively. The study that showed a 76% improvement [97] demonstrates how inventory management practices have evolved, significantly reducing inefficiencies and excessive stock.

On the other hand, the study that revealed a 20% improvement [104] suggests that even smaller advancements in inventory management can considerably impact a pharmacy's overall effectiveness. These findings highlight a progressive movement toward enhancing inventory practices in hospital pharmacies. By improving inventory management, these institutions are ensuring the availability of essential medications while simultaneously reducing waste and overall healthcare expenses. The focus on this aspect demonstrates a dedication to financial accountability and the delivery of excellent patient care.

Reduction in Medication Errors

Identified in the systematic review was a crucial theme concerning the significant decrease in medication errors found in hospital pharmacies, a key factor contributing to patient safety. Several studies [42,48-50,76-78,104] have reported remarkable reductions in medication error rates, ranging from 42% to 91%. These statistics highlight a fundamental shift toward safer pharmacy practices and improved medication handling and dispensing vigilance. For instance, one study [77] demonstrated a 91% reduction in errors, showcasing the potential for substantial improvements through targeted interventions and system-wide changes. Similarly, another study [104] showed an impressive 83% reduction, emphasizing the effectiveness of rigorous quality control and error prevention strategies. These studies consistently align with the global healthcare objective of minimizing patient harm by focusing on reducing medication errors. The methods to achieve these reductions likely involve advanced technology, staff training, process re-engineering, and enhanced quality assurance measures. These findings illustrate a dedicated effort within the hospital pharmacy field to tackle the critical issue of medication errors. This progress reflects advancements in technology and procedures and a more substantial commitment to providing patient-centered care and ensuring safety.

Increase in Satisfaction Rate

The systematic review underscored a significant finding: a noticeable improvement in satisfaction rates in hospital and pharmacy settings. This trend, which indicates higher satisfaction levels among patients and staff, can be observed in several studies [13,42,51,55,61,92], with increases ranging from 6% to 82%. These improvements substantially impact how pharmacy services are perceived in terms of quality and effectiveness. In particular, one study [55] reported a remarkable 82% increase in patient satisfaction, demonstrating how efforts to improve medication turnaround times, enhance communication, and implement patient-centric care approaches can significantly elevate satisfaction levels. Even when specific percentages were not provided [44], the focus on increased satisfaction rates shows how hospital pharmacies increasingly prioritize service quality and user experience. Another study found a 69% increase in satisfaction [51], further illustrating the successful adoption of strategies to enhance patient and staff satisfaction. These collective findings demonstrate that hospitals are placing importance on the technical aspects of pharmacy services and the subjective experiences of those who interact with these services. By doing so, hospitals are meeting and often surpassing their patients' and staff's expectations and needs, leading to a more positive healthcare environment.

*Improvements in* *Financial Performance and Reduction in Medication Expenditure (Cost)*

The systematic review has revealed a crucial finding concerning the practices of hospital pharmacies: they have been able to lower their medication costs and associated expenses significantly. This finding is of great importance for economic efficiency and healthcare sustainability. The studies [36,39,64,75,80,105] showed reductions in costs that range from 24% to an extraordinary 179%. For example, one study [64] reported a decrease of 179%, which is an exceptional improvement in cost efficiency. This improvement may have been achieved by implementing optimized procurement strategies, better inventory management, and waste reduction measures. Another study [75] found a reduction of 90%, which demonstrates the potential for significant savings in the pharmaceutical field. Even when specific percentages were not mentioned in certain studies [44], the consistent focus on reducing medication costs indicates that this is a priority for the entire industry.

Furthermore, the study [36] reported a reduction of 82%, further emphasizing the trend of implementing cost-effective measures without compromising the quality of care. These findings illustrate the targeted efforts made by hospital pharmacies to decrease medication-related expenses. Such efforts are crucial for the financial sustainability of healthcare institutions and can help make healthcare more accessible by relieving the financial burden on patients and the healthcare system.

Improvements in Clinical Pharmacy Activities and Service Outcomes

The evaluation showcased a noteworthy enhancement in clinical pharmacy operations, emphasizing clinical pharmacists' evolving role and impact in healthcare environments. Prominent progress can be observed in studies [56,57,88,96], which demonstrate significant improvements, ranging from a 50% increase in medication reconciliation to a remarkable 400% increase in ward areas with clinical coverage. Eliminating high-alert medication errors by 100% [57] exemplifies the crucial role played by clinical pharmacists in promoting patient safety and care. This reduction signifies a near-elimination of critical mistakes, highlighting the effectiveness of targeted interventions and increased attentiveness in pharmacy practice. Additionally, the 92% rise in medication reconciliation [88] and the 400% increase in ward areas with clinical coverage [96] indicate a substantial expansion in the range and impact of clinical pharmacy services. These enhancements are likely a result of closer integration between clinical pharmacists and patient care teams, thereby elevating the overall quality of healthcare provision. Even in studies without specific percentages available [99], the recurring emphasis on improvements in clinical pharmacy underscores the significance of joining the power of Lean and Six Sigma methodologies in advancing patient care. These findings suggest that hospital pharmacies are increasingly prioritizing clinical activities, indicating a shift toward a more patient-oriented approach. This shift is pivotal for enhancing patient outcomes, ensuring medication safety, and contributing to the overall effectiveness of healthcare systems.

Improvements in Prescription Checking and Intervention Success Rates

The systematic review has highlighted a critical aspect of hospital pharmacy practice: the notable improvements in prescription verification and intervention success rates. This topic is of utmost importance in ensuring the accuracy of medications and the safety of patients, as evidenced in studies [72,79], where documented improvements range from 25% to 100%. The outstanding 100% improvement reported in one study [79] is remarkable, signifying a scenario where prescription verification and interventions have reached optimal effectiveness. This level of improvement suggests that stringent standards and protocols have been successfully implemented, significantly reducing the risk of medication errors. In another study, a substantial 25% improvement in prescription verification [72] demonstrates progress in enhancing the reliability and precision of medication dispensing processes. This improvement can likely be attributed to a combination of factors, including the adoption of advanced technological systems, improved staff training, and more effective communication within the healthcare team. Even in studies where specific percentages were not provided [40], the inclusion of this topic indicates its significance in the broader context of enhancing pharmacy services. These findings underscore a growing emphasis on accuracy and excellence in pharmacy. By prioritizing improvements in prescription verification and intervention success rates, hospital pharmacies are directly contributing to enhanced patient safety and the overall quality of healthcare delivery.

Reduction in Workflow Interruptions

The systematic review emphasized the significance of reducing workflow interruptions in hospital pharmacy settings, a crucial factor in maintaining efficiency and ensuring continuous patient care. The research [34,71] demonstrated substantial decreases in workflow interruptions, with 60% and 75% reductions, respectively. A 60% decrease in workflow interruptions [34] indicates a noteworthy enhancement in the smooth functioning of pharmacy services. This decrease could be attributed to implementing streamlined procedures, adopting technological solutions for better workflow management, or changing staff communication and task assignment strategies. Particularly remarkable is the 75% reduction [71], which strongly emphasizes reducing distractions and interruptions that can hinder the pharmacy's efficiency. This significant decrease suggests a thorough reevaluation and redesign of workflow processes, potentially incorporating elements such as task automation, improved space utilization, or enhanced coordination between departments. These findings collectively demonstrate a dedicated effort to refine pharmacy operations, underscoring the critical role of uninterrupted workflow in maintaining high standards of pharmacy service. By minimizing disruptions, these pharmacies enhance their operational efficiency and significantly contribute to improved patient outcomes and overall healthcare quality.

Increase in Knowledge

The analysis incorporated a subject centered on the progress of understanding, specifically within the hospital pharmacy setting. As exemplified in the study [46], this subject demonstrates an 18% growth in understanding, highlighting the significance of ongoing learning and skill development in pharmacy practice. The 18% increase in understanding indicates a dedication to professional growth and education in the pharmacy industry. This improvement is likely a consequence of targeted training programs, educational workshops, or initiatives designed to keep pharmacy personnel updated with the most recent industry standards, medication information, and best practices. Such an emphasis on expanding understanding is crucial for ensuring that pharmacists and pharmacy technicians are adequately equipped to handle the complexities of modern healthcare. It emphasizes the worth placed on education to enhance service quality, improve patient safety, and keep up with the evolving demands of the healthcare sector. This focus on knowledge enlargement benefits the individuals involved and has a broader impact on the efficiency and effectiveness of pharmacy services as a whole. The drive to consistently enhance knowledge within the pharmacy sector demonstrates a forward-thinking approach, aimed at fostering excellence and innovation in healthcare.

Further insights and innovations from Lean Six Sigma in hospital pharmacy operations

Quality Improvement Initiatives

Six Sigma has been extensively utilized within the healthcare industry as an effective quality management system [115]. Quality improvement initiatives in hospital pharmacy aimed to enhance patient safety, optimize medication use, and improve healthcare outcomes. These initiatives streamlined processes, reduced errors, and ensured high-quality care. Evidence-based practices improved efficiency and effectiveness, fostering excellence and interprofessional collaboration. Effective communication and collaboration among healthcare professionals were vital. Tools like LSS and root cause analysis (RCA) identified causes of errors and developed strategies. Standardized operating procedures, technology solutions, and ongoing education improved safety and efficiency. Quality improvement initiatives also positively impacted financial performance, reducing errors and hospital stays, and improving satisfaction.

Competitive Advantage, Value of Service, and Safety Improvements

Operational excellence is crucial for any business entity to endure and thrive in the international marketplace. Numerous companies have implemented various continuous improvement ideologies to address this matter, including lean manufacturing, Six Sigma, total quality management, agile manufacturing, and more. Both lean manufacturing and Six Sigma have demonstrated exceptional outcomes [116]. Additionally, this review emphasized the significance of implementing lean practices and enhancing quality improvement in hospital pharmacy. These measures aimed to boost service delivery while also reducing costs associated with services, transportation, and the availability of affordable medication supplies. Ultimately, these efforts sought to increase patient satisfaction and gain a competitive advantage [106].

Staff Productivity and Motivation

Cleaner and more organized workspaces contributed to improved employee motivation and collaboration and fostered productivity and efficiency [117,118]. A tidy workspace helps employees stay focused, reduces distractions, and minimizes the time spent searching for tools and materials [59]. Additionally, an organized workspace promotes better time management and task prioritization, allowing employees to complete their tasks more effectively [82].

The shift in pharmacist activities from non-value-added to value-added activities significantly impacted productivity and satisfaction [98]. By eliminating tasks that did not directly contribute to the quality and efficiency of patient care, pharmacists focused more on providing valuable services such as medication counseling, medication therapy management, and drug therapy reviews [79]. This shift benefited patients by improving the quality of care received and allowed pharmacists to utilize their expertise and knowledge effectively. Lean management principles aim to eliminate waste, streamline processes, and improve efficiency in various industries, including healthcare. By equipping employees with the necessary knowledge and tools to identify and eliminate waste, organizations experienced significant improvements in productivity, quality, and customer satisfaction. Participants of lean management training programs learned techniques such as value stream mapping, 5S methodology, and continuous improvement, enabling them to contribute to the organization's overall success and performance [108].

Patient Engagement

The frequency of patient consultations was significantly increased as a strategic move to enhance the overall quality of healthcare delivery and to address patient concerns more promptly and effectively. This proactive approach led to improved health outcomes, as patients benefited from timely and personalized medical advice, guidance, and interventions [88]. In parallel, there was a rigorous implementation of medication safety measures aimed at preventing errors and minimizing adverse drug reactions, thereby ensuring a higher standard of patient care and safety.

Additionally, efforts were made to streamline the patient discharge process, a critical step in the patient care journey. This streamlining focused on creating more efficient and transparent procedures, facilitating smoother transitions from hospital to home or next-stage care, and significantly reducing the potential for confusion or misunderstandings about post-discharge care [57].

These strategic improvements in patient consultation frequency, medication safety, and discharge processes played a pivotal role in transforming the landscape of healthcare delivery. By focusing on these critical areas, healthcare providers were able to substantially enhance patient satisfaction and safety, thereby raising the overall standard of care [92]. This holistic approach to patient care not only addressed immediate health concerns but also contributed to a more patient-centric healthcare system.

Performance Metrics and Benchmarking

The utilization of KPIs significantly enhanced pharmacist productivity and, in turn, markedly improved patient care outcomes. This approach involved meticulously monitoring and assessing performance improvements within the medication dispensing cycle. Quantitating these improvements in sigma levels ensured the safety and efficiency of medication management [64,119]. Additionally, there was a concerted effort to optimize the allocation of pharmacists' time across a range of critical tasks. This strategic time management maximized their productivity and played a crucial role in delivering high-quality pharmaceutical services, ultimately contributing to better patient care and service delivery [74,82].

Cultivating patient-centric lean leadership in the hospital pharmacy operations

Leadership plays a crucial role in the management of operations, and the fundamental requirement for a sustainable implementation of lean in the healthcare sector is to secure the complete backing of the organization's leadership [120]. During the review process, an observation was made concerning the emergence of lean leadership in the pharmacy industry. This was accompanied by a gradual yet substantial understanding and implementation of lean philosophy principles. The fundamental concept of lean thinking guides the organization's vision to harmonize with the client's perspective. As this knowledge expands, the qualities of lean leadership emerge naturally and become inherent in the leadership approach. Lean pharmacy leaders aspire to create a culture that is unwaveringly focused on excellence, thus transforming the fundamental characteristics of leadership from traditionally authoritative and sporadic to collaborative, continuous, and empathetic toward the needs and experiences of the patients [121]. This shift is not only operational but also philosophical, guiding a comprehensive transformation that incorporates the principles of Lean and Six Sigma into the structure of hospital pharmacy operations, with the ultimate goal of achieving excellence in patient care. Table 3 compares conventional and lean approaches to pharmacy leadership, focusing on quality, standards, and engagement.

**Table 3:** Comparison between traditional and lean pharmacy leadership ‎

Traditional pharmacy leadership	Lean pharmacy leadership
Considers quality as a shared responsibility among several	Prioritizes quality above all else
Relies on statistical averages for measurement	Imposes and upholds strict standards
Performs evaluations monthly	Conducts daily assessments against benchmarks
Penalizes errors made	Commends the disclosure of errors
Influenced by isolated incidents	Guided by empirical data
Tends to suppress disagreements	Fosters an environment where disputes can be safely addressed
Directs organizational changes	Inspires collective action and decision-making
Demands that management upholds standards	Forges a connection with operational staff
Champions cost-saving measures	Drives a culture of quality enhancement and cost optimization
Exudes a charismatic aura	Demonstrates modesty
Concentrates on financial outcomes	Concentrates on the welfare of patients
Dominates communication down the hierarchy	Promotes patient engagement through direct interaction

The comparison between traditional and lean pharmacy leadership uncovered distinct approaches to managing pharmacy operations. Traditional pharmacy leadership is characterized by perceiving quality as a shared responsibility, relying on statistical averages, conducting evaluations every month, penalizing errors, being influenced by isolated incidents, suppressing disagreements, directing organizational changes, demanding management upholds standards, prioritizing cost-saving, focusing on financial outcomes, and adopting a top-down communication style. On the other hand, lean pharmacy leadership prioritizes quality as the ultimate importance, maintains stringent standards, performs daily assessments, commends the disclosure of errors, makes decisions based on empirical data, encourages open disputes, promotes collective decision-making, engages with operational staff, focuses on enhancing the quality and optimizing costs, demonstrates modesty, concentrates on patient welfare, and encourages patient engagement through direct interaction. This transition reflects the lean model as a more dynamic, inclusive, and patient-centered approach.

Lean Six Sigma DMAIC tools framework for hospital pharmacy

**Figure 11:**
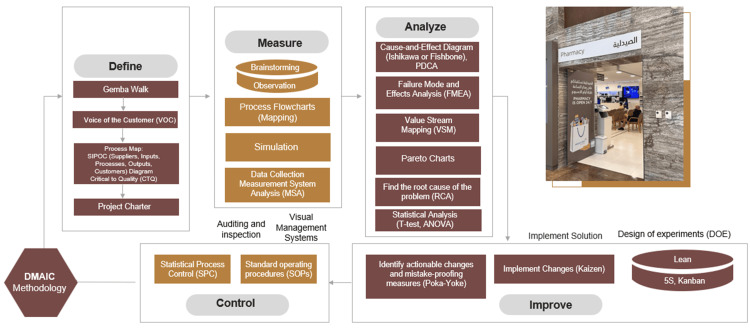
Lean Six Sigma DMAIC tools framework The template downloaded from SlideGeeks [122] with a valid subscription permission to all the website content Credit: Dr. Mohammed Sallam Gemba Walk: Japanese term meaning the physical workplace; PDCA: Plan-do-check-act; ANOVA: Analysis of variance; T-test: A statistical test used to compare the means of two groups; 5S: Sort, Set in order, Shine, Standardize, and Sustain; Kaizen: Japanese term meaning continuous improvement; Kanban: Japanese term meaning a visual work management system card; DMAIC: Define, measure, analyze, improve, and control

The systematic review undertaken for this research paper ended in the development of a condensed version of the LSS DMAIC tool framework, specially adapted for implementation in hospital pharmacy settings. This modification of the LSS DMAIC model is presented in Figure 11 as a concise and informative flow diagram [122]. This diagram serves as an important visual guide that effectively presents the sequential phases of defining, measuring, analyzing, improving, and controlling the strategic tools associated with each phase. As a result, it provides a structured approach to process improvement within hospital pharmacy environments.

In the defining phase, significant practices such as Gemba walks and the VOC are emphasized. Gemba walks involve visiting the work site to directly observe daily operations and identify areas for potential improvement. Meanwhile, the VOC technique focuses on comprehending the needs, expectations, and experiences of both patients and staff, which is crucial for establishing meaningful and patient-centered improvement goals.

The measuring phase employs tools like brainstorming sessions, enabling the collaborative generation of ideas, and the identification of key metrics for monitoring. Additionally, process flowcharts are utilized to visually represent existing workflows, with the aim of identifying inefficiencies and redundancies. These tools facilitate a comprehensive understanding of the current state of pharmacy operations.

During the analyzing phase, the use of cause-and-effect diagrams aids in determining the root causes of identified problems. These diagrams, supported by FMEA, enable a detailed exploration of potential failure points and their impacts on pharmacy processes, thereby laying the groundwork for targeted improvements.

In the improving phase, strategies such as Kaizen and the design of experiments are implemented. Kaizen emphasizes continuous, incremental improvements, focusing on enhancing efficiency and quality through manageable steps. Meanwhile, the design of experiments method allows for systematic experimentation to assess the effects of various changes on process outcomes, ensuring that improvements are data-driven and effective.

Last, the control phase incorporates statistical process control (SPC) and the establishment of standard operating procedures (SOPs). SPC is utilized to monitor process performance and sustain the gains achieved in previous phases, while SOPs guarantee consistency, safety, and compliance in daily pharmacy operations.

This comprehensive LSS DMAIC framework, as represented in the diagram, encapsulates the fundamental principles and methods of LSS, tailored specifically to address the unique needs and challenges of hospital pharmacies. By following this structured approach, hospital pharmacies are equipped to enhance their operational efficiency, service quality, and overall patient care. Consequently, this diagram and the accompanying explanation constitute a crucial component of the research paper, providing valuable insights and a practical roadmap for quality improvement initiatives in hospital pharmacy settings.

Study strengths and limitations

The review was characterized by a broad scope and rigorous PRISMA-guided methodology, including a standard review checklist and a four-phase flow diagram [27], providing a view of diverse management strategies with in-depth insights relevant to hospital pharmacy practice and policy-making. Incorporating the Six Sigma DMAIC cycle into the review process offered a potentially innovative approach, providing a well-defined procedure that integrated various established LSS methodological tools, thereby serving as valuable guidance for quality managerial decision-making in the pharmacy field [123].

However, the study faced limitations such as heterogeneity among the included studies and language bias, affecting the generalizability of findings. The evaluation solely encompassed written documents in English, which might lead to underestimation of research conducted in other languages. The primary focus of the review and its query was ‎based on observation, utilizing information to ascertain the LSS ‎tools employed in hospital pharmacies. Therefore, the quantity of objective verification to validate these findings was relatively limited, comprising solely of results derived from the studies that were considered. Additionally, the search algorithm for Lean and Six Sigma strategies implemented in hospital pharmacy services was limited to only the sections of title-abstract-keywords, which could constrain the results obtained. The industry's dynamic progression and the omission of grey literature may have influenced the review's completeness to some extent.

Recommendation for future studies

In future research, it is possible to explore further theories and methodologies related to operations management, specifically within hospital pharmacy. Investigating these possibilities can improve quality management in pharmacy and healthcare. Moreover, future evaluations might consider ‎adding objective factors and challenges for Lean and Six Sigma implementation. Eventually, there is a clear requirement for future empirical investigations to comprehensively examine and clarify the utilization of innovative management systems and applications driven by artificial intelligence and robotics [124].

## Conclusions

The author's primary contribution was gathering and analyzing substantial information about LSS tools and their impact on hospital pharmacy performance. This effort was achieved by thoroughly examining literature published between 2009 and 2023, accompanied by detailed assessment, deep analysis, and subsequent recommendations.

This systematic review figured out that implementing diverse Lean and Six Sigma management strategies was pivotal in enhancing the quality aspect of hospital pharmacy operations management. The review demonstrated the effectiveness of LSS methodologies in improving operational efficiency and attaining positive results in hospital pharmacies worldwide. It also provided insight into the dynamic interaction between Lean and Six Sigma approaches and their substantial impact on the quality of pharmacy operations and patient services. The findings emphasized the crucial role of context-specific strategies, CSFs, and the imperative for ongoing adaptation and innovation in management practices to align with the changing needs of healthcare. Moreover, the review provided quality-focused tools and techniques, CTQ elements, and KPIs for process enhancement.

This thorough consolidation has the potential to enhance the global comprehension of quality management in hospital pharmacies and become an integral reference for professionals and policymakers committed to improving pharmacy services and raising the standard of patient care.
